# Identification of Hypoglycemic Glycolipids from *Ipomoea murucoides* by Affinity-Directed Fractionation, In Vitro, In Silico and Dynamic Light Scattering Analysis

**DOI:** 10.3390/plants13050644

**Published:** 2024-02-26

**Authors:** Daniel Rosas-Ramírez, Roberto Arreguín-Espinosa, Sonia Escandón-Rivera, Adolfo Andrade-Cetto, Gerardo Mata-Torres, Ricardo Pérez-Solís

**Affiliations:** 1 Departamento de Química de Biomacromoléculas, Instituto de Química, Universidad Nacional Autónoma de México, Av. Universidad 3000, Circuito Exterior s/n, Coyoacán, Ciudad Universitaria, Mexico City 04510, Mexico; arrespin@unam.mx (R.A.-E.); ricardo.perez@itsatlixco.edu.mx (R.P.-S.); 2 Departamento de Biología Celular, Facultad de Ciencias, Universidad Nacional Autónoma de México, Av. Universidad 3000, Circuito Exterior s/n, Coyoacán, Ciudad Universitaria, Mexico City 04510, Mexico; aac@ciencias.unam.mx (A.A.-C.); gerardom.torres@ciencias.unam.mx (G.M.-T.); 3 Departamento de Ingenieria Mecatrónica, Tecnológico Nacional de México, Instituto Tecnológico Superior de Atlixco, Heliotropo 1201, Unidad 8 Norte Nueva Xalpatlaco, Vista Hermosa, Atlixco 74218, Mexico

**Keywords:** *Ipomoea murucoides*, resin glycosides, glycolipids, glucose-6-phosphatase inhibitor, *α*-glucosidase inhibitor, molecular docking, dynamic light scattering

## Abstract

In the pursuit of identifying the novel resin glycoside modulators glucose-6-phosphatase and *α*-glucosidase enzymes, associated with blood sugar regulation, methanol-soluble extracts from the flowers of *Ipomoea murucoides* (cazahuate, Nahuatl), renowned for its abundance of glycolipids, were employed. The methanol-soluble extracts were fractionated by applying the affinity-directed method with glucose-6-phosphatase enzymes from a rat’s liver and *α*-glucosidase enzymes from its intestines. Mass spectrometry and nuclear magnetic resonance were employed to identify the high-affinity compound as a free ligand following the release from the enzymatic complex. Gel permeation through a spin size-exclusion column allowed the separated high-affinity molecules to bind to glucose-6-phosphatase and *α*-glucosidase enzymes in solution, which led to the identification of some previously reported resin glycosides in the flowers of cazahuate, where a glycolipid mainly structurally related to murucoidin XIV was observed. In vitro studies demonstrated the modulating properties of resin glycosides on the glucose-6-phosphatase enzyme. Dynamic light scattering revealed conformational variations induced by resin glycosides on *α*-glucosidase enzyme, causing them to become more compact, akin to observations with the positive control, acarbose. These findings suggest that resin glycosides may serve as a potential source for phytotherapeutic agents with antihyperglycemic properties.

## 1. Introduction

The *Ipomoea genus*, a member of the Convolvulaceae family, boasts a global distribution and is renowned for its medicinal properties, particularly attributed to resin glycosides found in various plant tissues, including flowers, leaves, and roots. Notably, *Ipomoea* species have played pivotal roles in pre-Hispanic cultures for food (*Ipomoea batatas*), religious rituals (*Ipomoea violacea*), and medicinal applications (*Ipomoea purga*) [1]. One such species, *Ipomoea murucoides* Roem. et Schult., native to Mexico, holds significance within the arborescent plant group. Historically, the Aztecs utilized it for preventing hair loss [2,3], while its flowers and bark served medicinal purposes, such as staunching bleeding and acting as an antidote to scorpion stings and snake bites [4,5].

In the context of *I. murucoides*, approximately twenty-eight resin glycosides have been identified, the most significant amount being in the flowers with nineteen murucoidins (I-XIX), in addition to stoloniferin I and pescaprein III, previously isolated in other plants [2,3,4,5]. In addition to murucoidins isolated in the flowers, five murucins and two murucoidins were isolated from the bark exudate [6,7]. Resin glycosides, characterized by their high molecular weight and amphipathic nature, consist of a hydrophilic oligosaccharide chain and a lipophilic aglycone, typically comprising monohydroxylated fatty acids of fourteen or sixteen carbon atoms. This aglycone often forms an intramolecular ester with the oligosaccharide chain, which is partially acylated by some organic acids [6,7].

Beyond their chemical complexity, glycolipids within the Convolvulaceae family exhibit diverse biological activities. Of particular interest is their demonstrated potential as antibacterial agents against multi-drug-resistant (MDR) strains of *Staphylococcus aureus* [3,4,8]. The antimicrobial activity in MDR by the glycolipids may be closely related to the metabolism of glucose of *S. aureus* since glucose-6-phosphate (G6P) is a common alternative carbon source for some pathogenic bacteria MDR, such as *S. aureus*, which can utilize alternative carbon sources under glucose-limiting conditions. It is essential for its fitness in the host environment during the infectious process [9,10], and hence these glycolipids are also possibly related to other protective functions in the plant.

In addition to acting as an antimicrobial activity in MDR, Convolvulaceae glycolipids have been identified as a source of potent *α*-glucosidase inhibitors [11]. These glycolipids, with a pseudotretrasaccharide structure reminiscent of acarbose (therapeutic control), have exhibited stronger *α*-glucosidase inhibitory activity than the therapeutic positive control [11]. Consequently, this study aims to assess the resin glycosides in cazahuate flowers as inhibitors of rat hepatic glucose-6-phosphatase and rat intestinal *α*-glucosidases. The goal is to establish glycolipids as potential phytotherapeutic agents with modulating properties on enzymatic activity. Furthermore, the interaction between these glycolipids and yeast *α*-D-glucosidase is explored, focusing on conformational variations studied through dynamic light scattering (DLS). DLS, known for its quick, label-free, and non-destructive nature, provides valuable insights into the size and aggregation state of proteins, nucleic acids, complexes, viruses, or virus-like particles (VLPs) [12,13].

## 2. Results

### 2.1. LRESI-MS Study of the Methanolic Extract from the Flowers of Cazahuate

Given the prior identification of nineteen glycolipids in *I. murucoides* flowers [2,3,4,5], a low-resolution electrospray ionization mass spectrometry (LRESI-MS) study was initiated on the methanolic extract. Conducted at concentrations analogous to those used in enzyme–ligand complex cleavage processes, this study revealed five prominent signals in the resin glycosides region. Notably, signals at *m/z* 1143 (64%) and *m/z* 1289 (13%) were identified, likely corresponding to five murucoidins through their cationized molecules [M + Na]^+^ [3,4]. Specifically, these signals may represent murucoidins XII or XIII (molecular weight: 1120 amu) and murucoidins VI, X, or XI (molecular weight: 1266 amu). Additionally, signals at *m/z* 1045 (28%), 1197 (38%), and 1375 (9%) were observed, not aligning with reported resin glycosides in *I. murucoides*. Since this sample was not incubated with an enzyme, all peaks’ relative abundance (%) can directly indicate the amount of each cation relative to the amount present for its non-cationized analytes in the tested sample (based on the most intense peak arbitrary set at 100%). 

### 2.2. Affinity-Directed Fractionation with α-Glucosidases and the Methanolic Extract from Flowers of Cazahuate

Given the high affinity of glycolipids for *α*-D-glucosidase from *Saccharomyces cerevisiae*, this enzyme was selected, which allows us to compare the compounds observed in the enzymes of therapeutic interest. In addition to knowing the content of metabolites in the methanolic extract by LR-ESI-MS, experiments were also carried out with the incubation buffer, showing the background noise; with *α*-glucosidase, showing the enzyme peaks; and, finally, the therapeutic control, the free ligand (acarbose: *m*/*z* 668 [M + Na]^+^) of the *α*-glucosidase–acarbose complex after elution in spin-exclusion column (SEC) and released by acid denaturing conditions [11]. Affinity screening with yeast *α*-D-glucosidase revealed signals at *m/z* 1029 (25%), 1127 (95%), 1175 (100%), and 1191 (33%) in the resin glycosides region. These four signals may correspond to eight of the nineteen murucoidins previously isolated in the *I. murucoides* flowers through their cationized molecules [M + Na]^+^ [2,3,4], murucoidins XV or XVI with a molecular weight of 1006 amu (1029 [M + Na]^+^), murucoidin XIV with a molecular weight of 1104 amu (1127 [M + Na]^+^), murucoidin III or stoloniferin I with a molecular weight of 1152 amu (1175 [M + Na]^+^), and murucoidin IV, V, or VIII with a molecular weight of 1168 amu (1191 [M + Na]^+^). In addition, a signal at *m*/*z* 1185 (14%) was observed in the resin glycosides region, which does not correspond to the resin glycosides reported in *I. murucoides*. 

Now that it is known that the glycolipids present in *I. murucoides* flowers have an affinity for the yeast *α*-D-glucosidase enzyme, affinity experiments were extended to rat intestine *α*-glucosidase. Affinity screening allowed the detection of signals at *m*/*z* 1127 (11%), 1175 (13%), and 1203 (18%) in the resin glycosides region. These three signals correspond to four of the nineteen murucoidins previously isolated through their cationized molecules [M + Na]^+^ [2,4], murucoidin XIV with a molecular weight of 1104 amu (1127 [M + Na]^+^), and murucoidin III or stoloniferin I with a molecular weight of 1152 amu (1175 [M + Na]^+^). In addition, the signal observed at *m*/*z* 1203 does not correspond to any resin glycoside reported in *I. murucoides*.

### 2.3. Molecular Docking

Docking studies were carried out to improve our understanding of the interaction of selected compounds, observed in affinity-directed fractionation with yeast and intestinal *α*-glucosidases and the methanolic extract from flowers of cazahuate, inside the catalytic site of MAL12 and MGAM-C, which were selected as the template for molecular modeling. Homology modeling of MAL12 was first performed to predict its three-dimensional structure, which was not determined by using the crystal structure of *α*-glucosidase from *Bacillus cereus* [11]. Additionally, the crystal structure of the human maltase-glucoamylase (MGAM-C, the C-terminal subunit) was selected for modeling to propose binding modes for the analyzed resin glycosides inside the catalytic site of this enzyme [11]. 

**Figure 1:**
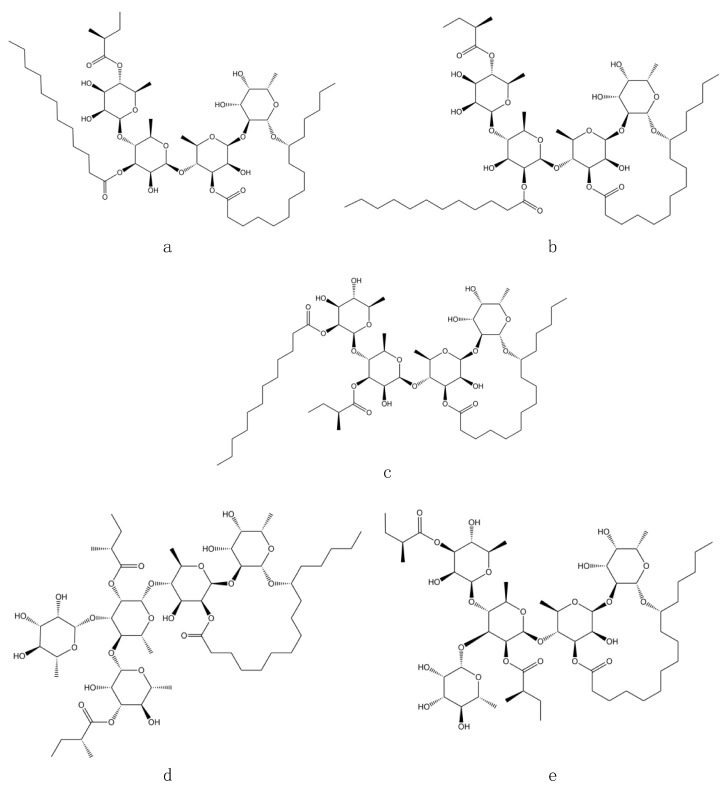
Structure of glycolipids selected for docking studies: (**a**) murucoidin XIV; (**b**) pescaprein V; (**c**) pescaprein VI; (**d**) murucoidin III; (**e**) stoloniferin I.

Ligand–receptor molecular models were also calculated for acarbose (used as a positive control) to test the adapted protocol with both *α*-glucosidase templates. Acarbose fits well in the catalytic pocket of both analyzed enzymes and showed hydrogen bonding interactions between the amino acid residues HIS279 (2.08 Å), GLN322 (2.00 Å), and ARG312 (2.15 Å) and MAL12, while the binding modes inside the catalytic site of MGAM-C corresponded to TYR1251 (1.93 Å), GLN1372 (1.75 Å), ARG1377 (2.11 Å), GLN1561 (2.07 Å), and GLY1588 (2.17 Å) [11]. Ligand–receptor molecular models were also calculated for the glycolipids that were observed in the affinity screening with yeast and intestinal *α*-glucosidase enzymes. Three glycolipids were identified in affinity studies that have a similar molecular weight to that previously observed in the flowers of *I. murucoides*: murucoidin XIV with a molecular weight of 1104 amu (1127 [M + Na]^+^) and murucoidin III and stoloniferin I with a molecular weight of 1152 amu (1175 [M + Na]^+^). In addition to these glycolipids, the tetrasaccharides diastereoisomers with molecular formula C_52_H_92_O_19_ pescapreins V and VI, previously reported from *Ipomoea pescapre* [14] with a molecular weight of 1104 amu (1127 [M + Na]^+^) and similar structure to murucoidin XIV, were considered for docking studies (Table 1). Therefore, it was decided to use these five glycolipids for docking studies (Figure 1). 

### 2.4. Affinity-Directed Fractionation with Glucose-6-Phosphatase (G6Pase) and the Methanolic Extract from Cazahuate Flowers

**Figure 2:**
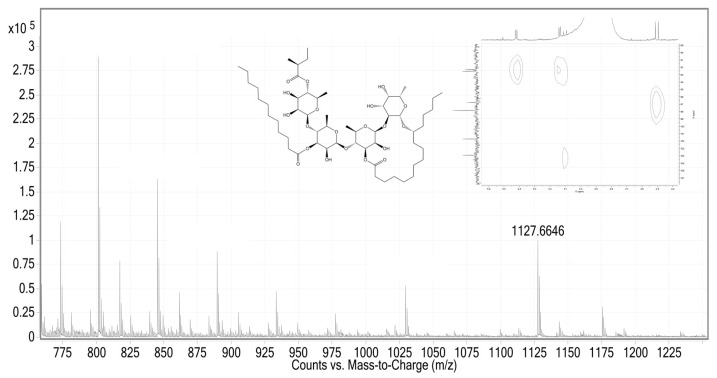
HR-ESI mass spectra in positive mode and HSQC-NMR were obtained from the fraction extract with hepatic G6Pase enzyme.

The application of affinity screening, involving the incubation of the methanolic extract with G6Pase and the subsequent employment of the Size-Exclusion Chromatography/Electrospray Ionization Mass Spectrometry (SEC/ESI-MS) protocol [11,15], facilitated the detection of signals at *m/z* 1029.5568 (5%), 1127.6646 (11%), and 1175.6113 (3%) on a high-resolution (HR) spectrometer (Figure 2). These signals correspond to five of the nineteen murucoidins previously isolated in *I. murucoides* through their cationized molecules [M + Na]^+^ [2,4]: murucoidins XV and XVI with a molecular weight of 1006 amu (1029 [M + Na]^+^), murucoidin XIV with a molecular weight of 1104 amu (1127 [M + Na]^+^), and murucoidin III and stoloniferin I with a molecular weight of 1152 amu (1175 [M + Na]^+^). 

### 2.5. In Vitro Hepatic Glucose-6-Phosphatase (G6Pase) Activity

**Figure 3:**
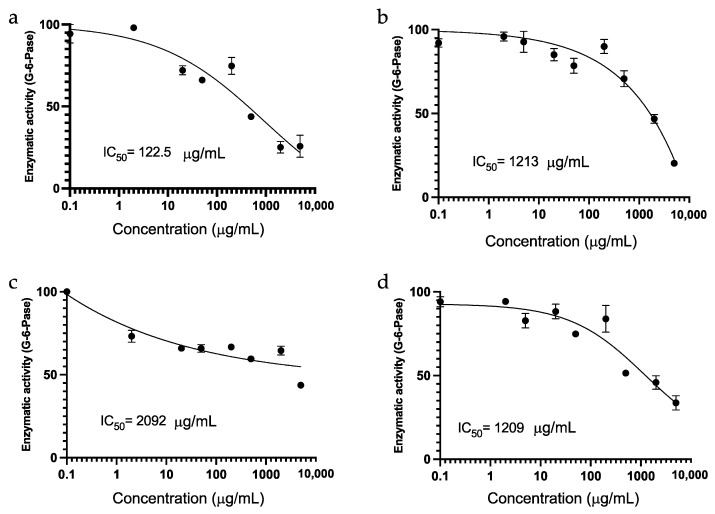
Concentration-response inhibition assays of hepatic G6Pase of (**a**) chlorogenic acid as a positive control; (**b**) methanol soluble extract of flowers from *I. murucoides*; (**c**) fraction extract with yeast *α*-D-glucosidase enzyme; and (**d**) fraction extract with hepatic G6Pase enzyme.

An in vitro investigation was undertaken, involving the G6Pase enzyme, the methanol extract, and the fractions derived from the affinity processes of hepatic G6Pase and yeast *α*-D-glucosidase enzymes (Figure 3). Chlorogenic acid served as the positive control, exhibiting an IC_50_ of 122.5 μg/mL (Figure 3a). The methanol-soluble extract from the flowers of *I. murucoides* demonstrated an IC_50_ of 1213 μg/mL (Figure 3b). The two spin fractions exhibited an IC_50_ of 2092 μg/mL for the fraction extracted with yeast α-D-glucosidase enzyme (Figure 3c) and an IC_50_ of 1209 μg/mL for the fraction extracted with the G6Pase enzyme (Figure 3d). 

### 2.6. Dynamic Light Scattering (DLS) with Yeast α-D-Glucosidase and the SEC Protocol

**Figure 4:**
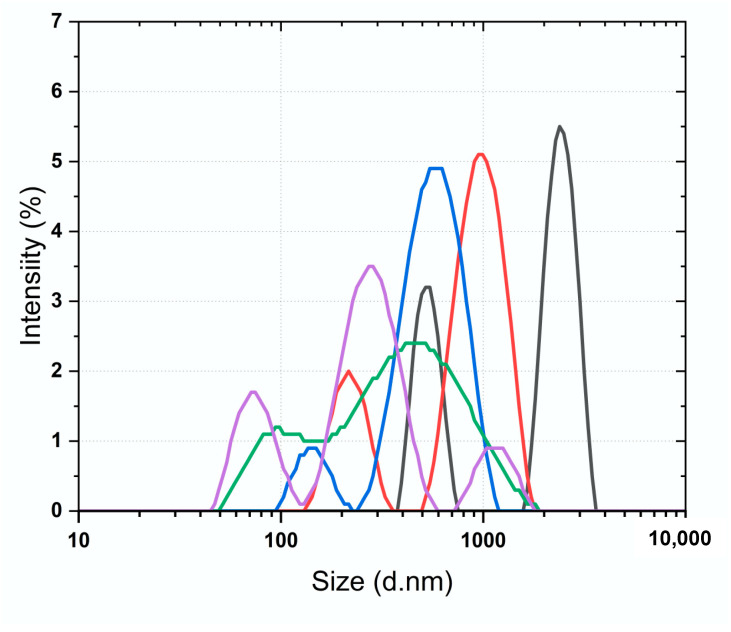
Dynamic light scattering (DLS) size distribution by intensity of (black) yeast *α*-D-glucosidase enzyme; (red) acarbose as a positive control; (blue) yeast *α*-D-glucosidase enzyme after SEC protocol; (green) acarbose–yeast *α*-D-glucosidase enzyme complex after SEC protocol; (purple) methanol soluble extract–yeast *α*-D-glucosidase enzyme complex after SEC protocol.

A DLS investigation was conducted, involving the yeast *α*-D-glucosidase enzyme, the fraction obtained from the methanolic extract incubation with yeast *α*-D-glucosidase enzyme, and acarbose as a positive control (Figure 4). Particle size determination, a direct measure of conformational variations in a protein, was carried out using DLS to ascertain particle size distributions and hydrodynamic sizes in the nano-to-micrometer range (nm–μm) in liquid dispersions. Initially, measurements were taken for the yeast *α*-D-glucosidase enzyme alone, the enzyme after the SEC process, and the reference therapeutic agent, yielding Z-average values of 1422.6 ± 58.15, 438.8 ± 29.98, and 624.9 ± 15.59 d.nm, respectively (Figure 4). Subsequently, the SEC process was applied to the methanolic extract and acarbose incubated with yeast *α*-D-glucosidase enzyme, resulting in Z-average values of 243.7 ± 9.47 and 260.7 ± 61.68 d.nm, correspondingly (Figure 4).

## 3. Discussion

### 3.1. Affinity-Directed Fractionation with α-Glucosidases and the Methanolic Extract from Flowers of Cazahuate

Comparing the glycolipids that were observed in the affinity screening with yeast enzyme and the intestinal enzyme, two signals were found to be similar: murucoidin XIV with a molecular weight of 1104 amu (1127 [M + Na]^+^) and murucoidin III or stoloniferin I with a molecular weight of 1152 amu (1175 [M + Na]^+^). But these results do not allow us to know which glycolipids were bound to the yeast enzyme from before the screening. Therefore, the murucoidins IV, V, XIV, XVIII, XIX, XX, and stoloniferin I were tested to know their potential as yeast *α*-D-glucosidase inhibitors, but none of these glycolipids showed any inhibitory activity. Since a glycolipid previously inactive to inhibit the yeast enzyme was observed in the affinity studies coupled to ESI-MS, the affinity studies coupled with one- and two-dimensional (2D) nuclear magnetic resonance (NMR) were selected to identify the glycolipid structure that shows high affinity for yeast *α*-D-glucosidase enzymes. Two-dimensional NMR studies only allowed us to determine that the oligosaccharide nucleus of the glycolipid with high affinity to the yeast *α*-D-glucosidase enzyme belongs to operculinic acid C, a tetrasaccharide with one fucose unit (^1^H, dd, *d*_H_ 4.48; *d*_C_ 96.76) and three rhamnose units (^1^H, dd, *d*_H_ 5.08, *d*_C_ 103.92; ^1^H, dd, *d*_H_ 5.12, *d* 92.28; ^1^H, dd, *d*_H_ 5.40, *d*_C_ 92.57), previously identified in *Merremina mammosa, Ipomea operculata, I. batatas*, and in *I. murucoides* [1].

Most of these oligosaccharides are glycosidic derivatives of (11*S*)-11-hydroxyhexadecanoic (jalapinolic) and (11*S*)-11-hydroxytetradecanoic (convolvulinolic) acids, which form a macrocyclic ester through lactonization (intramolecular esterification) with the carboxylic group of the aglycone. C-1 jalapinolic acid is observed at 175.89 (C, Jla-1). In the HSQC spectrum, this C-1 is not observed to have a correlation with ^1^H signals, which indicates that its high-affinity glycolipid indeed forms intramolecular esterification. The presence of the macrocyclic structure is an essential requirement for biological activity [1]. In addition to being esterified by jalapinolic or convolvulinolic acid, the glycolipids generally present their oligosaccharide nuclei as acylated, forming esters with volatile and non-volatile fatty acids. The most frequent volatile fatty acids in species of the genus *Ipomoea* are tiglic (tga), isobutyric (iba), methylbutyric (mba), nilic (nla), and cinnamic (cna). The fatty acid previously identified in murucoidin III, XIV, and stoloniferin I is mba, which esterifies murucoidin III and stoloniferin I with two mba residues. Murucoidin XIV, in addition to one unit of mba, is esterified by a high-molecular-weight fatty acid, dodecanoic acid. High-molecular-weight fatty acids characterized in species of the genus *Ipomoea* include hexanoic (hexa), octanoic (octa), decanoic (deca), and dodecanoic (dodeca) acids. However, the NMR spectra do not show the characteristic signals of the mentioned compounds. The chemical nature and structural complexity of this mixture of glycolipids constitute an obstacle that makes it challenging to characterize the molecular structure of the glycolipid with high affinity to the yeast *α*-D-glucosidase enzyme. Therefore, it was decided to conduct docking studies to establish which of the previously identified glycolipids related to molecular weights seen in affinity studies could be related to the observed activity.

### 3.2. Molecular Docking

**Figure 5:**
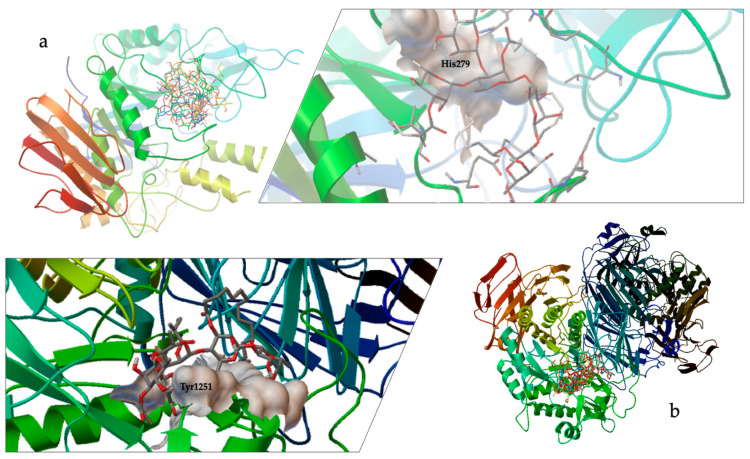
Docking results using the structural model of (**a**) *α*-D-glucosidase (MAL12) from *S. cerevisiae*, which comprises the catalytic site of the enzyme (surface in white) and stoloniferin I; and (**b**) C-terminal subunit of human maltase-glucoamylase (MGAM), which comprises the catalytic site of the enzyme (surface in white) and stoloniferin I.

This analysis predicted that all docked compounds formed hydrogen bonds with amino acid residues that are part of the catalytic site, primarily with HIS279 in MAL12 (Figure 5a, Table 1) and preserved catalytic residues around TYR1251 in MGAM (Figure 5b, Table 1), which is involved in the catalytic substrate specificity of this protein [11,15]. These results indicate that the structure of the pentasaccharide stoloniferin I is the best conformation of the glycolipids selected for the inhibition of the two enzymes under study (Table 1), resulting in a lower concentration value for the theoretical Ki for MAL12 (244 mM) and for MGAM (4.28 mM). The binding conformation and orientation in the enzyme catalytic site for stoloniferin I provoked a more significant steric impediment at the receptor’s surface, making it challenging to access the catalytic pocket (Figure 5).

### 3.3. Affinity-Directed Fractionation with Glucose-6-Phosphatase (G6Pase) and the Methanolic Extract from Cazahuate Flowers

As affinity studies coupled to HRESI-MS offer limited structural insights, parallel investigations were conducted, utilizing 2D NMR techniques (Figure 2) to elucidate the structure of the glycolipid exhibiting high affinity for G6Pase. These studies revealed that the oligosaccharide nucleus of the glycolipid with high affinity to the enzyme aligns with operculinic acid C, a tetrasaccharide characterized by one fucose unit and three rhamnose units. This oligosaccharide core is notably present in murucoidin XIV, previously identified in *M. mammosa, I. operculata, I. batatas*, and *I. murucoides* [1]. However, it is imperative to note that reference signals of the high-affinity glycolipid to the enzyme were not discernible in the NMR spectra [16]. These findings mirror those observed with *α*-glucosidase enzymes. However, in this instance, no coupling studies were undertaken; instead, an in vitro study was conducted with the hepatic G6Pase enzyme.

### 3.4. In Vitro Hepatic Glucose-6-Phosphatase (G6Pase) Activity

Significantly, the fraction obtained in affinity screening with the G6Pase enzyme displayed the most inhibition of the hepatic G6Pase enzyme. The notorious difference in IC_50_ values between the fractions obtained with the *α*-D-glucosidase enzyme implies the distinct nature of the isolated glycolipids. Notably, the primary glycolipid observed, murucoidin XIV, has previously exhibited antimicrobial activity against SA-1199B (MIC 32 μg/mL), a norfloxacin-resistant strain overexpressing the NorA multi-drug resistance (MDR) efflux pump [4]. The demonstrated activity of this glycolipids with G6Pase enzyme, coupled with its reported antimicrobial activity in MDR, suggests a potential correlation with glucose metabolism. Given that glucose-6-phosphate (G6P) serves as a common alternative carbon source for certain pathogenic bacteria, including *S. aureus* [9,10], it is plausible that these glycolipids fulfill additional protective functions in the plant. Nevertheless, further studies are imperative to comprehensively understand these potential roles. As far as we know, our current work is the first report of modulatory activity of glycolipids on G6Pase.

### 3.5. Dynamic Light Scattering (DLS) with Yeast α-D-Glucosidase and the SEC Protocol

This study involved establishing methodologies for the discernment of biological activity beyond traditional methods, such as chromogenic assays, where disadvantages that include sensitivity to turbidity and interference from substances and auto-fluorescent compounds (UV quenchers) represent factors that significantly affect the results of tests based on light detection. As a formal examination of this approach, a DLS investigation was conducted, wherein yeast *α*-D-glucosidase was designated as the enzyme. Acarbose was used as a positive control (IC_50_, 370.99 ± 3.92 μM). The outcomes suggest a comparable interaction between acarbose and the yeast *α*-D-glucosidase enzyme, mirroring the interaction between the glycolipids of *I. murucoides* and the yeast α-glucosidase enzyme. This observation is evident in the reduction in size in the enzyme–ligand complex. Notably, the change in size between the enzyme alone and the enzyme after the SEC process signifies a conformational variation process induced by SEC. However, the conformational variations induced by the extract and the reference inhibitor is more pronounced. In silico studies provided additional insights, indicating that resin glycosides from the flowers of cazahuate engage with *S. cerevisiae α*-D-glucosidase (MAL12) at catalytic site residues (Figure 5). This interaction closely resembles the binding pattern observed with the pseudotetrasaccharide acarbose.

## 4. Materials and Methods

### 4.1. General Experimental Procedure

Low-resolution ESI-MS data were measured in a Bruker Daltonics Esquire 6000 ESI ion trap mass spectrometer (Bruker, Billerica, MA, USA). Nitrogen was used as both nebulizing and drying gas. Mass spectra were acquired over 50–1500 *m*/*z* in 10 s/scan using electrospray ionization with a capillary voltage set to 4 kV. High-resolution ESI-MS was measured in a coupled liquid chromatography system with single quadruple mass spectrometry and flight time (HPLC-EM-SQ-TOF Model G6530BA, Agilent Technologies, Inc., Santa Clara, CA, USA). ^1^H (400 MHz) and ^13^C (125.7 MHz) NMR experiments were conducted on a Bruker Avance III instrument.

### 4.2. Plant Material and Extracts

The sample analyzed consisted of dried flowers (100 g) of *I. murucoides* collected in Mexico City in October 2020 (19.3015384, −99.1616579). A voucher for the species was deposited at the Herbarium of Science Faculty, UNAM. The dried and powdered plant material was subjected to extraction with methanol through a maceration process. The solution was filtered once the extraction time was over, and the solvent was removed under reduced pressure. After removal of the solvents, a dark brown syrup (5 g) was obtained.

### 4.3. Affinity-Directed Fractionation

The affinity-directed fractionation methods used in the present investigation were previously described in [11,15]. Gel permeation chromatography was performed with a spin column packed with polyacrylamide (BioRad Laboratories, Hercules, CA, USA). The gel and samples were prepared in a solution of 0.1 M sodium phosphate buffer (pH 6.8). Aliquots (10 μL, in triplicates) of the methanol-soluble extract (200 μg/mL), as well as of chlorogenic acid (positive control for G6Pase) and acarbose (positive control for *α*-glucosidases) at a concentration of 2000 μM, were independently incubated for 5 min with 20 μL of the enzymes’ stock solution: 0.9 units/mL of yeast *α*-D-glucosidase in 100 μM of buffer solution; 40 μg of rat intestinal *α*-glucosidase in 100 μM of buffer solution; and G6Pase was performed using microsomal fractions suspended in buffer (40 mM imidazole, 250 mM sucrose, pH 7) [11,15,17]. Upon loading the test samples at the top of the spin-exclusion column (SEC), the mixtures were eluted by centrifugation at RCF 42,985× *g* for 4 min; then, the eluate (200 μL), corresponding to the solvent front and containing the enzyme–inhibitor complex, was collected and treated as previously described by denaturing acidic conditions [11,15]. The samples were analyzed by direct flow injection on an ion trap ESI mass spectrometer and NMR experiments.

### 4.4. Glucose-6-Phosphatase Assay

The concentration–response inhibition assays of hepatic G6Pase activity were tested through colorimetric assays that measure phosphate formation [18]. G6Pase assay was performed using microsomal fractions, which were obtained through differential centrifugation from livers of starved Wistar rats [17]. The microsomes were suspended in buffer (40 mM imidazole, 250 mM sucrose, pH 7), to which chlorogenic acid (control) or methanol-soluble extract were added at different concentrations from 2 µg/mL to 5000 µg/mL. The reaction started with 80 mM glucose-6-phosphate (G6P) and was incubated at 20 °C for 20 min. Later, stop solution (0.42% ammonium molybdate in 1 N H_2_SO_4_, 10% SDS, and 10% ascorbic acid) was added, and the mixture was incubated at 45 °C for 20 min. The absorbances were obtained at 830 nm. IC_50_ values were calculated by plotting concentration–response curves to find the best-fitting regression model (linear or non-linear). Analysis was performed in GraphPad Prism version 7.00 (GraphPad Software, La Jolla, CA, USA).

### 4.5. Molecular Docking

Docking was carried out with Auto Dock 4.2 software (The Scripps Research Institute, La Jolla, CA, USA) using the default parameters as previously described [11,15]. The molecular docking was performed with a model built by homology with *Bacillus cereus α*-glucosidase (1UOK.PDB) for the amino acid sequence of MAL12 from *S. cerevisiae*, which was retrieved from the UniProt protein resource data bank (accession code P5334) with the following preserved catalytic residues: His111, Asp205, Glu276, His348, and Asp349. The crystal structure resolution of the C-terminal subunit of MGAM (3TOP.PDB) was also used as the maltase template for molecular modeling. HyperChem 8 was necessary to calculate the energy-minimized form with geometric optimization for all ligands. AutoDock 4.2 tools prepared all files by adding polar hydrogen atoms. They merged non-polar hydrogens to the enzyme structures and computed Gasteiger charges for the molecular model of analyzed compounds as previously described for acarbose [11,15]. The entire system was subjected to surface scanning and refined docking. The theoretical inhibition constant (Ki) was performed by molecular docking with Auto Dock 4.2 software and was obtained from the binding energy (ΔG) using the formula: Ki = exp(ΔG/RT), where R is the universal gas constant (1.985 × 10^−3^ kcal mol^−1^ K^−1^) and T is the temperature (298.15 K).

### 4.6. Dynamic Light Scattering

Dynamic light scattering (DLS) was used to determine particle size using the Zetasizer Nano equipment (Malvern, Malvern, UK). Aliquots (10 μL) of the methanol-soluble extract (200 μg/mL) as well as of acarbose (positive control for *α*-glucosidases) at a concentration of 2000 μM were independently incubated for 5 min with 20 μL of the enzyme stock solution [11]. Upon loading the test samples at the top of the spin-exclusion column (SEC), the mixtures were eluted by centrifugation at RCF 42,985× *g* for 4 min; then, the eluate (200 μL), corresponding to the solvent front and containing the enzyme–inhibitor complex, was collected and used for DLS experiments. Milli-Q water and a low-volume quartz cell were used as dispersants at room temperature. Samples were performed in triplicate and processed in four 5 min runs [12,13].

### 4.7. Chromogenic Inhibition Assay of α-D-Glucosidase (EC 3.2.1.20) from S. cerevisiae

Aliquots of 10 μL of acarbose or test compounds in the concentration range of 0.2 to 2 mg/mL (in triplicate) were incubated for 5 min with 20 μL of the enzyme stock solution: 0.9 units/mL of *α*-D-glucosidase (EC 3.2.1.20) from *S. cerevisiae* (Sigma-Aldrich, St. Louis, MO, USA) and 100 μM solution of sodium phosphate buffer. After incubation, 10 μL of substrate [*p*-nitrophenyl-*α*-D-glucopyranoside, 5 mM in 0.1 M sodium phosphate buffer] was added and incubated for 35 min at 37 °C; then, the absorbances were determined. Regression analysis was used to calculate the concentration required to inhibit the enzyme activity by 50% (IC_50_), using the equation *V* = (*A*_100_)/[1 + (*I*/IC_50_)*^S^*, where *V* is the percentage of inhibition, *A*_100_ is the maximum inhibition, *I* is the inhibitor concentration, IC_50_ is the concentration required to inhibit the activity of the enzyme by 50%, and *S* is the cooperative degree [11,11].

## 5. Conclusions

This investigation unveils novel prototypes of enzyme modulators through affinity studies, employing a microscale technique for protein purification with size exclusion chromatography in a centrifugation column. This method, through a combination of analytical techniques, including LR and HR ESI-MS, two-dimensional NMR, molecular docking, and in vitro assays involving glucose-6-phosphatase (G6Pase) activity, enabled the identification of glycolipids that act as inhibitors of hepatic G6Pase and intestinal *α*-glucosidase enzymes. 

Affinity studies with yeast and rat intestine *α*-glucosidases, and G6Pase enzymes, discern five of the previously reported nineteen murucoidins in *I. murucoides* flowers, with notable emphasis on murucoidin XIV. However, the mass spectra also hint at the existence of additional signals, suggesting the possibility of discovering novel glycolipids, necessitating further exploration using alternative separation techniques. Molecular docking studies predicted strong interactions between selected glycolipids and the catalytic sites of *α*-glucosidases (MAL12 and MGAM-C), indicating potential inhibitory activity, and DLS investigations highlighted conformational variations induced by the glycolipids and demonstrated comparable interactions of glycolipids with yeast *α*-D-glucosidase to that of the reference inhibitor for *α*-glucosidase enzymes, acarbose.

In conclusion, this multi-faceted investigation provides a comprehensive understanding of the glycolipids from *I. murucoides* flowers, shedding light on their structural diversity, enzyme interactions, and potential medicinal properties. Glycolipids with structural similarities to murucoidin XIV obtained from the affinity process exhibited affinity for G6Pase, and the fraction obtained from this affinity process demonstrated inhibition of hepatic G6Pase activity. This is the first report of glycolipids’ modulatory activity on G6Pase. It is pertinent to highlight that only murucoidins, closely related to the flowers of *I. murucoides*, were identified. This underscores the utility of the affinity technique in determining the chemotaxonomic identity of the plant material under study.

## Figures and Tables

**Table 1:** *α*-Glucosidase theoretical inhibition of glycolipid selected for docking study.

Compound	Formula /MW	MAL12		MGAM	
		Theoretical Ki	Hydrogen Bond	Theoretical Ki	Hydrogen Bond
Murucoidin XIV	C_57_H_100_O_20_/1104	2.86 mM	ASN241 HIS279 GLN322	12.1 mM	GLY1588
Pescaprein V	C_57_H_100_O_20_/1104	1.23 mM	HIS279	346 mM	GLN1254
Pescaprein VI	C_57_H_100_O_20_/1104	304 mM	HIS279	118 mM	GLN1254 TRP1369 GLN1372 ARG1377
Murucoidin III	C_56_H_96_O_24_/1152	2.4 mM	HIS279	677 mM	ARG1377 THR1586
Stoloniferin I	C_56_H_96_O_24_/1152	244 mM	HIS279	4.28 mM	SER1366 ASP1370 GLN1372 ARG1377
Acarbose	C_25_H_43_NO_18_/645	51.4 μM	HIS279 GLN322 GLU304 ARG312	35.7 μM	TYR1251 GLN1372 ARG1377 GLN1561 GLY1588

## Data Availability

Data are contained within the article and its Supplementary Materials.

## Supplementary Materials

The following supporting information can be downloaded at: https://www.mdpi.com/article/10.3390/plants13050644/s1.

## References

[1] Pereda-Miranda R., Rosas-Ramírez D., Castañeda-Gómez J., Kinghorn A.D., Falk H., Kobayashi J. (2010). Resin Glycosides from the Morning Glory Family. Progress in the Chemistry of Organic Natural Products.

[2] Chérigo L., Pereda-Miranda R. (2006). Resin Glycosides from the Flowers of *Ipomoea murucoides*. J. Nat. Prod..

[3] Chérigo L., Pereda-Miranda R., Fragoso-Serrano M., Jacobo-Herrera N., Kaatz G.W., Gibbons S. (2008). Inhibitors of Bacterial Multidrug Efflux Pumps from the Resin Glycosides of *Ipomoea murucoides*. J. Nat. Prod..

[4] Chérigo L., Pereda-Miranda R., Gibbons S. (2009). Bacterial Resistance Modifying Tetrasaccharide Agents from *Ipomoea murucoides*. Phytochemistry.

[5] Corona-Castañeda B., Chérigo L., Fragoso-Serrano M., Gibbons S., Pereda-Miranda R. (2013). Modulators of antibiotic activity from *Ipomoea murucoides*. Phytochemistry.

[6] León I., Enríquez R.G., Nieto D.A., Alonso D., Reynolds W.F., Aranda E., Villa J. (2005). Pentasaccharide Glycosides from the Roots of *Ipomoea murucoides*. J. Nat. Prod..

[7] León I., Vera L.G., del Rio-Portilla F., Aranda E., Hernández-Velazquez V.M., Guevara Fefer P., Montiel E., Castillo P., Salinas Sanchez D.O. (2013). Resin Glycosides from *Ipomoea murucoides* and their effects on growth of *Spodoptera frugiperda*. J. Entomolju..

[8] Corona-Castanñeda B., Pereda-Miranda R. (2012). Morning glory resin glycosides as modulators of antibiotic activity in multidrug-resistant Gram-negative bacteria. Planta. Med..

[9] Yang Y., Sun H., Liu X., Wang M., Xue T., Sun B. (2016). Regulatory mechanism of the three-component system HptRSA in glucose-6-phosphate uptake in *Staphylococcus aureus*. Med. Microbiol. Immunol..

[10] Wang H., Wang M., Xu X., Gao Z., Xu Z., Zhang Q., Li H., Yan A., Kao R.Y.T., Sun H. (2021). Silver’s multi-target mode of action against *Staphylococcus aureus* endows it with the capability to combat antibiotic resistance. Nat. Commun..

[11] Rosas-Ramírez D., Pereda-Miranda R., Escandón-Rivera S., Arreguín-Espinosa R. (2020). Identification of *α*-Glucosidase Inhibitors from *Ipomoea Alba* by Affinity-Directed Fractionation-Mass Spectrometry. Rev. Bras. Farmacogn..

[12] Lorber B., Fischer F., Bailly M., Roy H., Kern D. (2012). Protein Analysis by Dynamic Light Scattering: Methods and Techniques for students. Biochem. Mol. Biol. Educ..

[13] Mirasol F. (2021). Stability Testing of Protein Therapeutics Using DLS. BioPharm. Int..

[14] Escobedo-Martínez C., Pereda-Miranda R. (2007). Resin Glycosides from *Ipomoea pes-caprae*. J. Nat. Prod..

[15] Rosas-Ramírez D., Escandón-Rivera S., Andrade-Cetto A., Arreguín-Espinosa R. (2020). Glucose-6-Phosphatase and *α*-Glucosidase Inhibitors from *Smilax moranensis* Roots Identified by Affinity-Directed Fractionation. Rev. Bras. Farmacogn..

[16] Pereda-Miranda R., Bautista E., Martínez-Fructuoso L., Fragoso-Serrano M. (2023). From relative to absolute stereochemistry of secondary metabolites: Applications in plant chemistry. Rev. Bras. Farmacogn..

[17] Espinoza-Hernández F., Andrade-Cetto A., Escandón-Rivera S., Mata-Torres G., Mata R. (2021). Contribution of fasting and postprandial glucose-lowering mechanisms to the acute hypoglycemic effect of traditionally used *Eryngium cymosum* F. Delaroche. J. Ethnopharmacol..

[18] Arion W.J. (1989). Measurement of intactness of rat liver endoplasmic reticulum. Methods Enzymol..

