# Supplementary material for: Identification of Hypoglycemic Glycolipids from Ipomoea murucoides by Affinity-Directed Fractionation, In Vitro, In Silico and Dynamic Light Scattering Analysis
Source: Plants (Basel). 2024 Feb 26;13(5):644. doi: 10.3390/plants13050644 (PMC10934653; doi:10.3390/plants13050644)

Identification of Hypoglycemic Glycolipids from *Ipomoea murucoides* by Affinity-Directed Fractionation, In Vitro, In Silico and Dynamic Light Scattering Analysis

Daniel Rosas–Ramírez[1,*], Roberto Arreguín–Espinosa[1], Sonia Escandón–Rivera[2,*], Adolfo Andrade–Cetto[2], Gerardo Mata–Torres[2], and Ricardo Pérez–Solís[1,3]

[1] Departamento de Química de Biomacromoléculas, Instituto de Química, Universidad Nacional Autónoma de México, Av. Universidad 3000, Circuito Exterior S/N, Coyoacán, Ciudad Universitaria, Mexico City, 04510, Mexico

[2] Departamento de Biología Celular, Facultad de Ciencias, Universidad Nacional Autónoma de México, Av. Universidad 3000, Circuito Exterior S/N, Coyoacán, Ciudad Universitaria, Mexico City 04510, Mexico

[3] Departamento de Ingenieria Mecatrónica, Tecnológico Nacional de México, Instituto Tecnológico Superior de Atlixco, Heliotropo 1201, Unidad 8 Norte Nueva Xalpatlaco, Vista Hermosa, Atlixco, 74218, Mexico

* Correspondence: dgrosas@unam.mx (D.R.-R.); soniaer@ciencias.unam.mx (S.E.-R.); Tel: +52- 5556224516 (D.R.-R.)

Table of Contents

Figure S1. The ^{1}H NMR spectrum of metanolic extract from flower of *Ipomoea murucoides* in CD_3OD (400 MHz).
Figure S2. The ^{1}H NMR spectrum of fraction extract with yeast α-D-glucosidase enzyme in CD_3OD (400 MHz).
Figure S3. The ^{13}C NMR spectrum of fraction extract with yeast α-D-glucosidase enzyme in CD_3OD (125 MHz).
Figure S4. The DEPT90 spectrum of fraction extract with yeast α-D-glucosidase enzyme in CD_3OD (125 MHz).
Figure S5. The DEPT135 spectrum of fraction extract with yeast α-D-glucosidase enzyme in CD_3OD (125 MHz).
Figure S6. The COSY spectrum of fraction extract with yeast α-D-glucosidase enzyme in CD_3OD.
Figure S7. The TOCSY spectrum of fraction extract with yeast α-D-glucosidase enzyme in CD_3OD.
Figure S8. The NOESY spectrum of fraction extract with yeast α-D-glucosidase enzyme in CD_3OD.
Figure S9. The HSQC spectrum of fraction extract with yeast α-D-glucosidase enzyme in CD_3OD.
Figure S10. The HMBC spectrum of fraction extract with yeast α-D-glucosidase enzyme in CD_3OD.
Figure S11. The ^{1}H NMR spectrum of fraction extract with rat intestine α-glucosidase in CD_3OD (400 MHz).
Figure S12. The ^{13}C NMR spectrum of fraction extract with rat intestine α-glucosidase enzyme in CD_3OD (125 MHz).
Figure S13. The HSQC spectrum of fraction extract with rat intestine α-glucosidase enzyme in CD_3OD.
Figure S14. The HMBC spectrum of fraction extract with rat intestine α-glucosidase enzyme in CD_3OD.
Figure S15. The ^{1}H NMR spectrum of fraction extract with hepatic G6Pase enzyme in CD_3OD (400 MHz).
Figure S16. The ^{13}C NMR spectrum of fraction extract with hepatic G6Pase enzyme in CD_3OD (125 MHz).
Figure S17. The DEPT90 spectrum of fraction extract with hepatic G6Pase enzyme in CD_3OD (125 MHz).
Figure S18. The DEPT135 spectrum of fraction extract with hepatic G6Pase enzyme in CD_3OD (125 MHz).
Figure S19. The HSQC spectrum of fraction extract with hepatic G6Pase enzyme in CD_3OD.

Figure S1. The ^{1}H NMR spectrum of metanolic extract from flower of *Ipomoea murucoides* in CD_3OD (400 MHz).

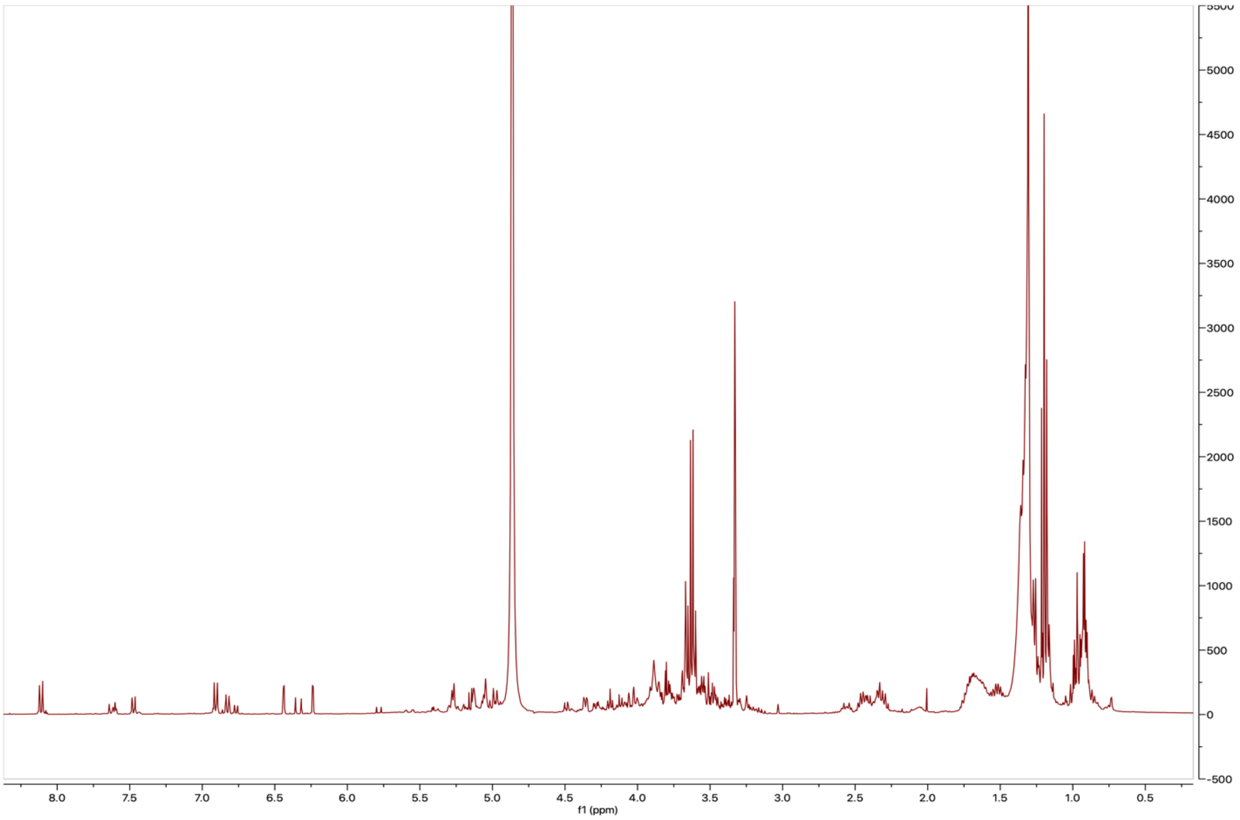

Figure S2. The ^{1}H NMR spectrum of fraction extract with yeast α-D-glucosidase enzyme in CD_3OD (400 MHz).

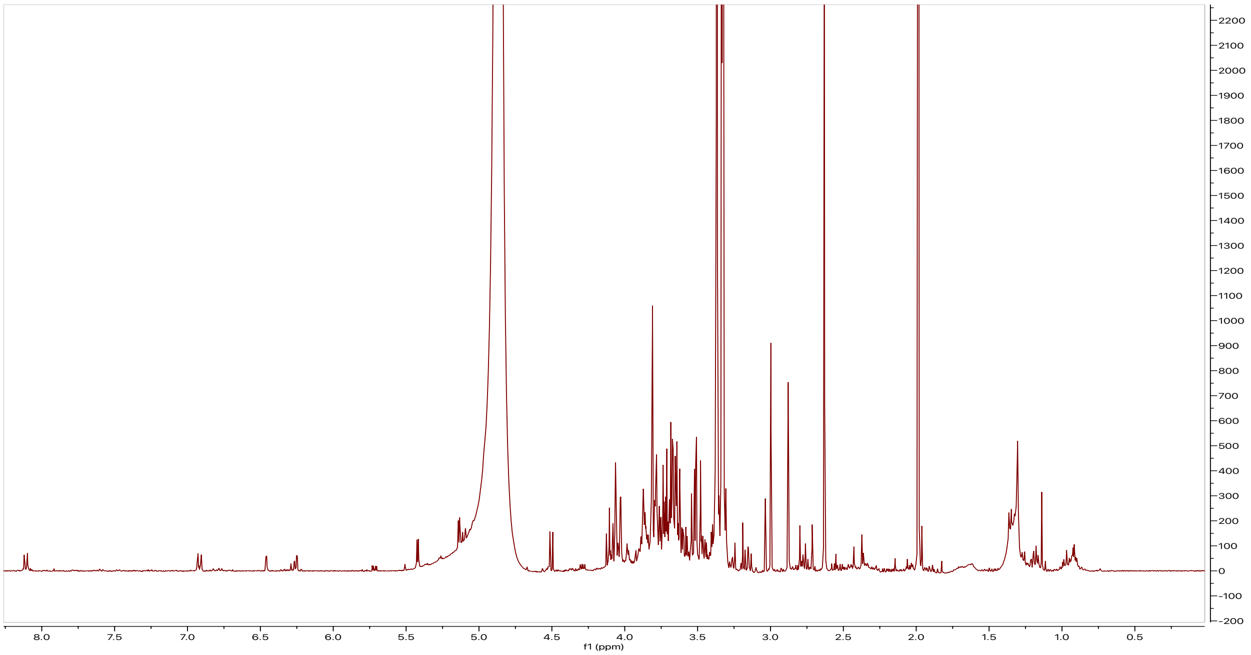

Figure S3. The ^{13}C NMR spectrum of fraction extract with yeast α-D-glucosidase enzyme in CD_3OD (125 MHz).

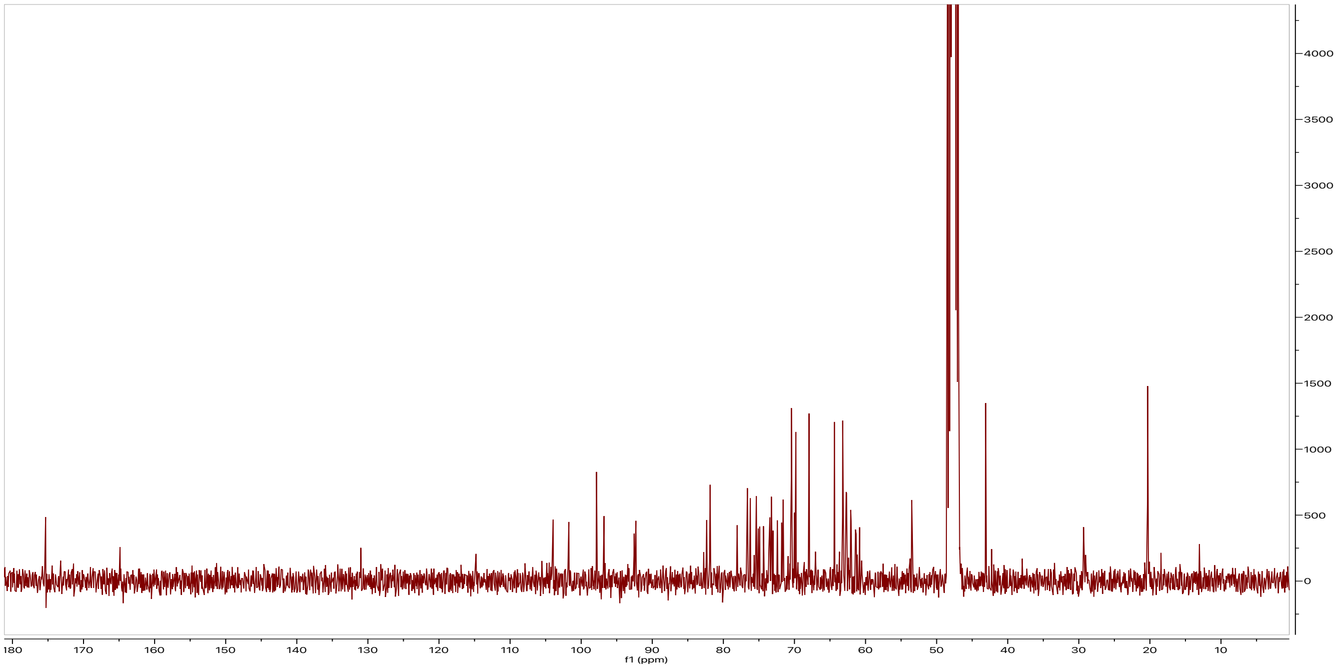

Figure S4. The DEPT90 spectrum of fraction extract with yeast α-D-glucosidase enzyme in CD_3OD (125 MHz).

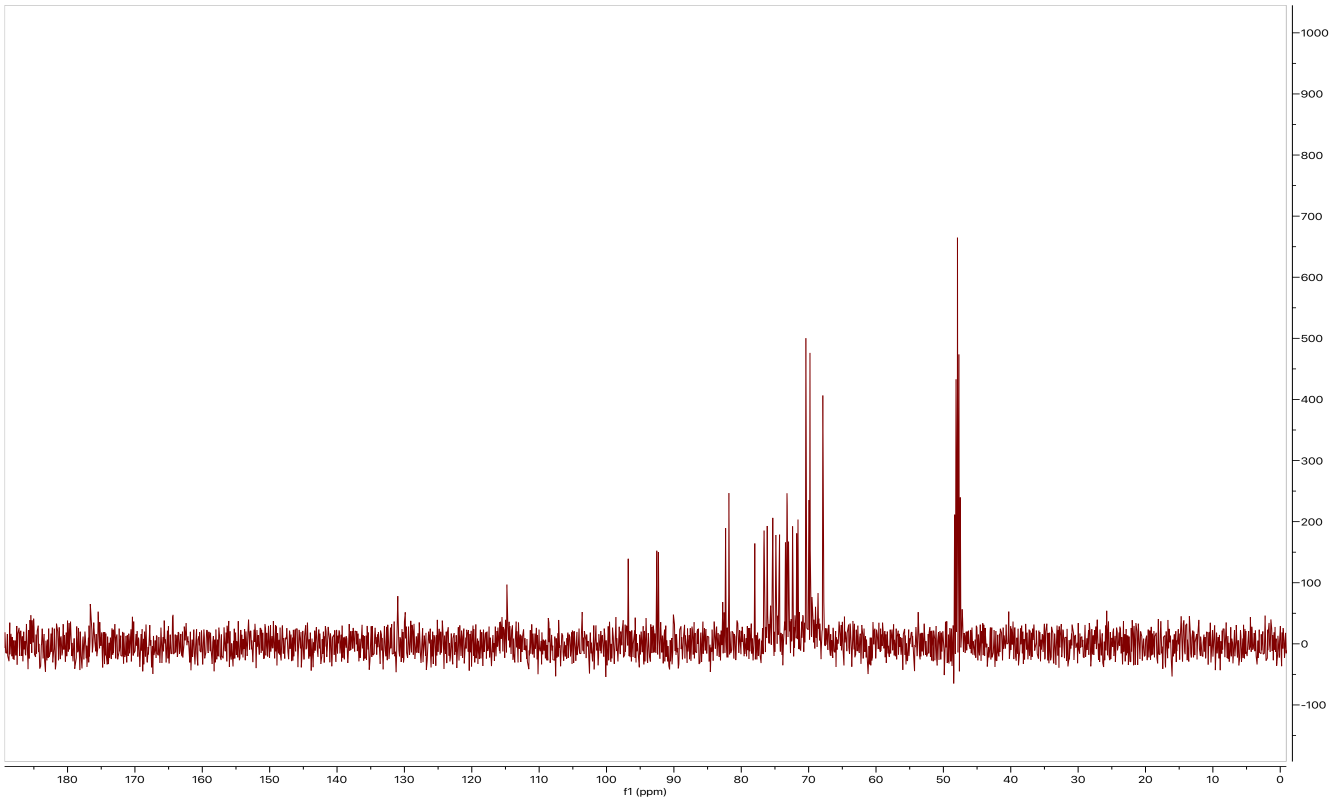

Figure S5. The DEPT135 spectrum of fraction extract with yeast α-D-glucosidase enzyme in CD_3OD (125 MHz).

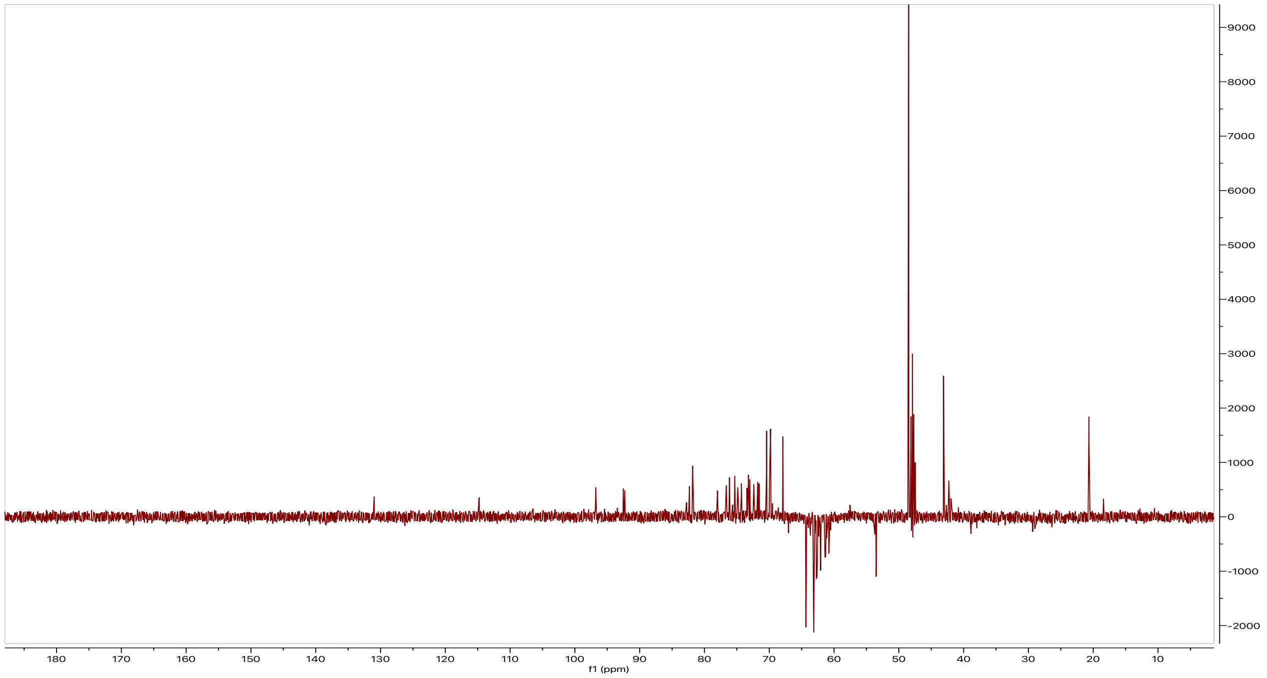

Figure S6. The COSY spectrum of fraction extract with yeast α-D-glucosidase enzyme in CD_3OD.

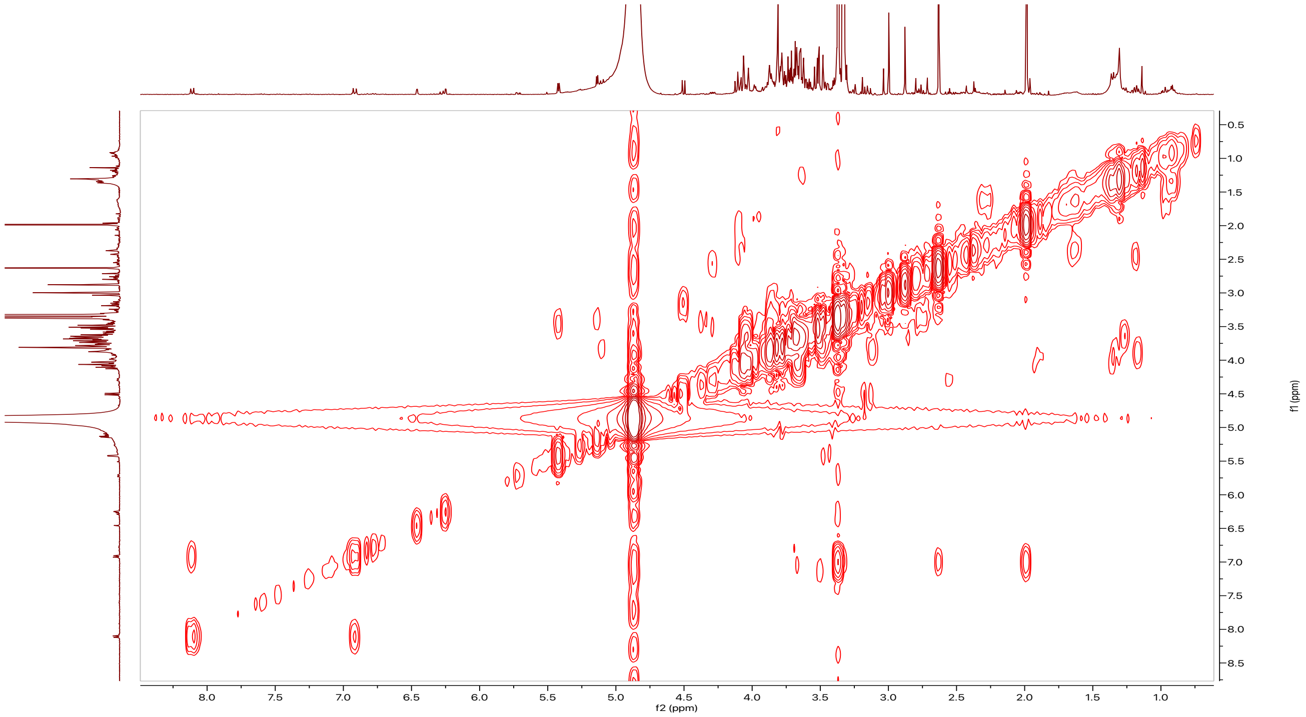

Figure S7. The TOCSY spectrum of fraction extract with yeast α-D-glucosidase enzyme in CD_3OD.

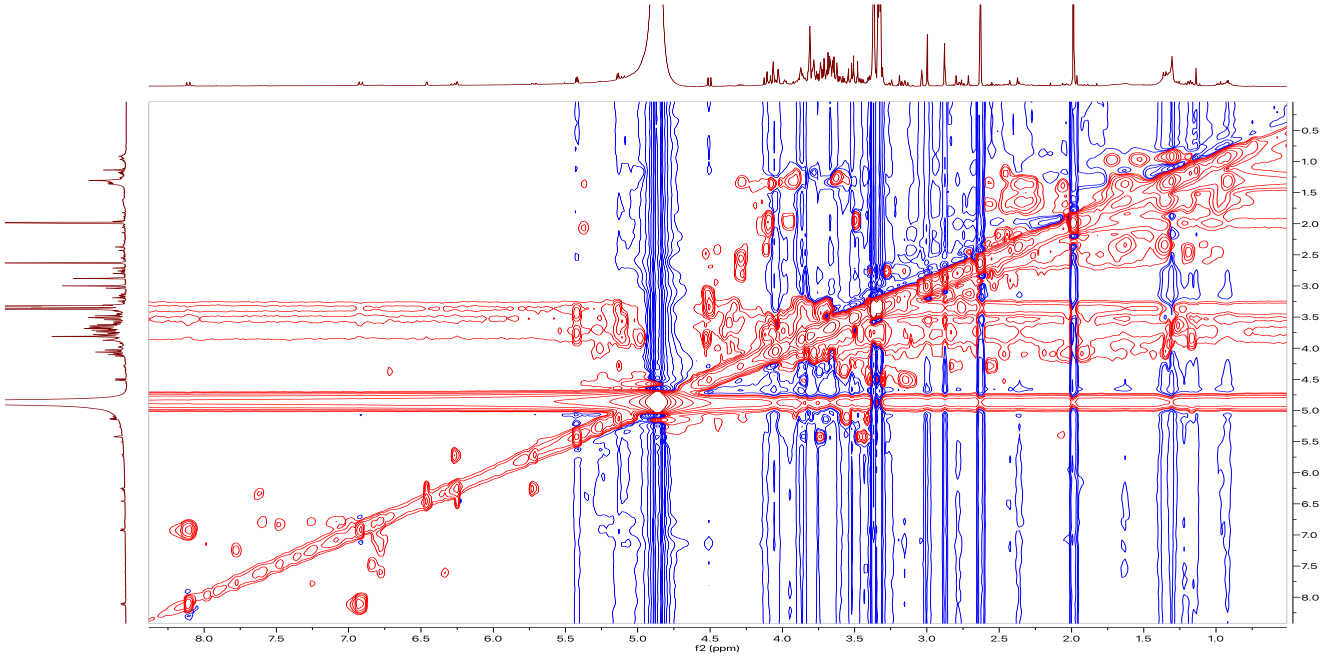

Figure S8. The NOESY spectrum of fraction extract with yeast α-D-glucosidase enzyme in CD_3OD.

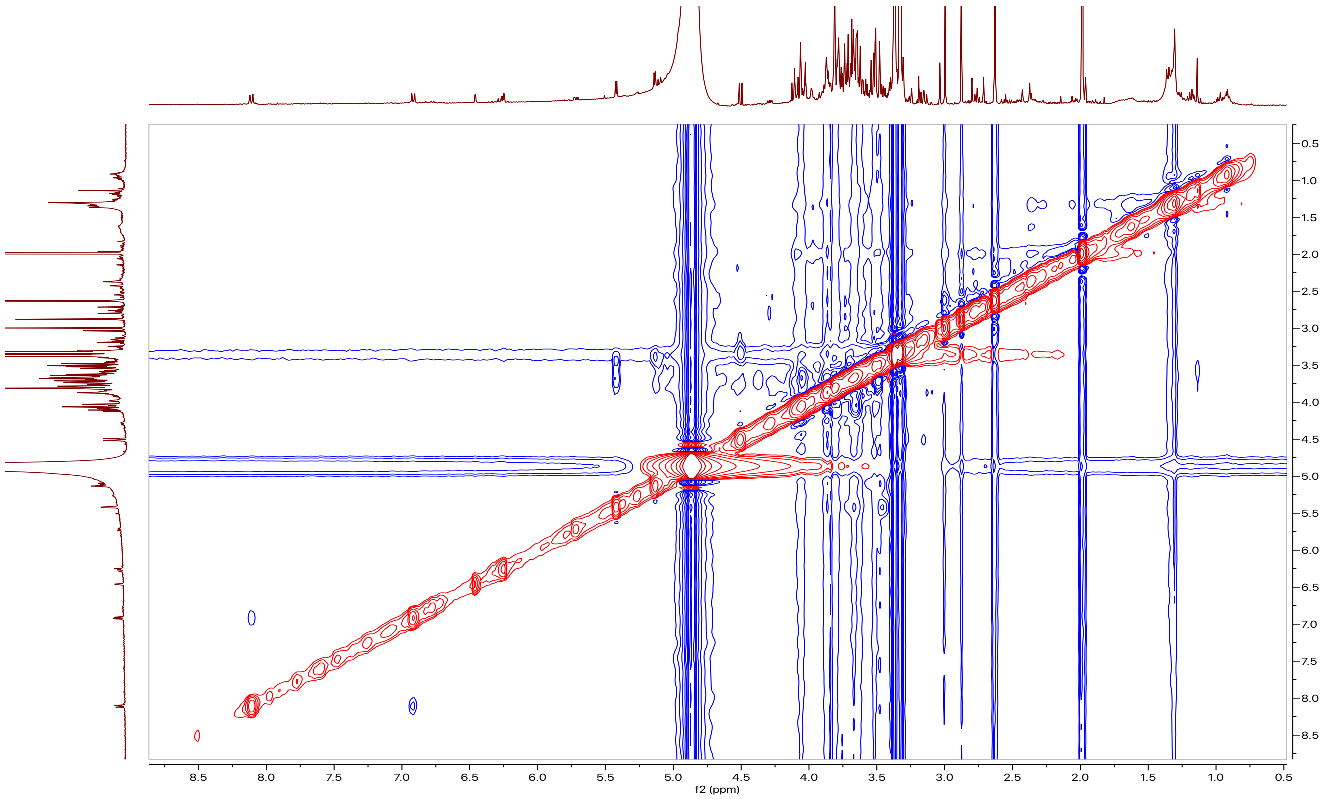

Figure S9. The HSQC spectrum of fraction extract with yeast α-D-glucosidase enzyme in CD_3OD.

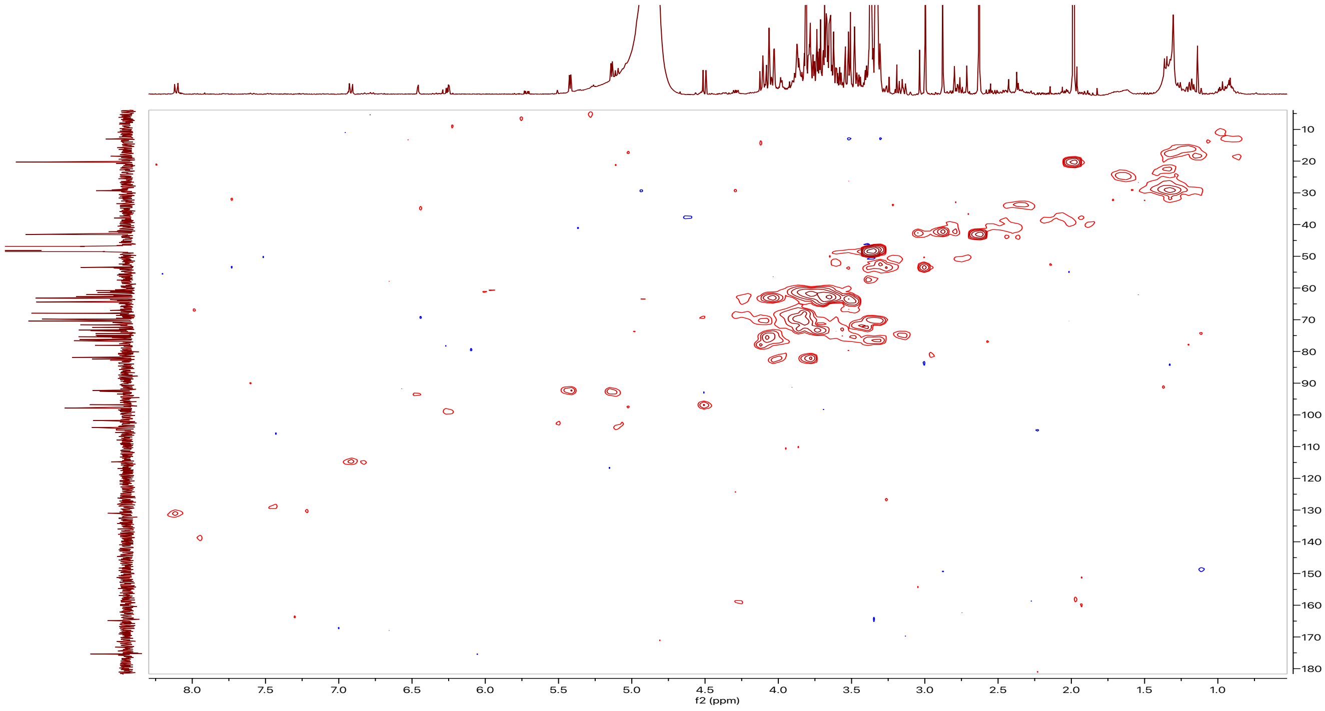

Figure S10. The HMBC spectrum of fraction extract with yeast α-D-glucosidase enzyme in CD_3OD.

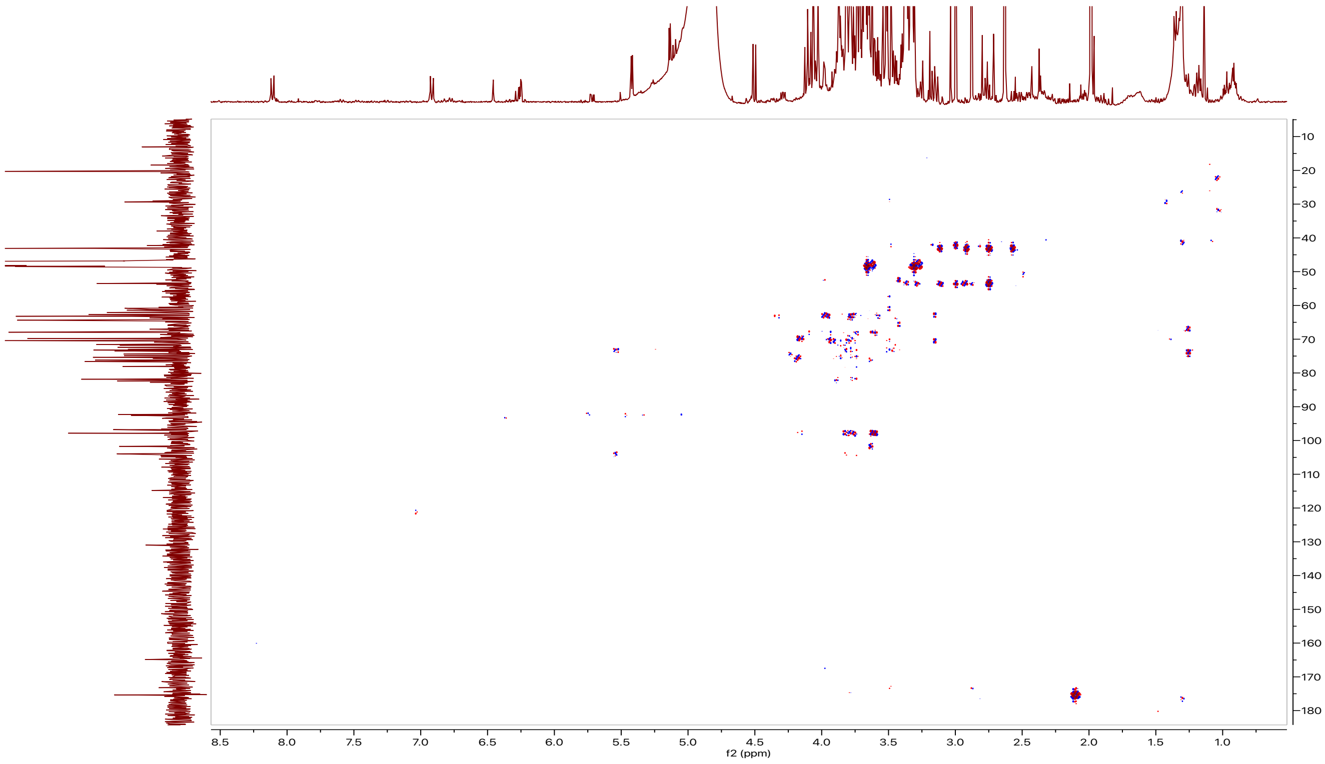

Figure S11. The ^{1}H NMR spectrum of fraction extract with rat intestine α-glucosidase in CD_3OD (400 MHz).

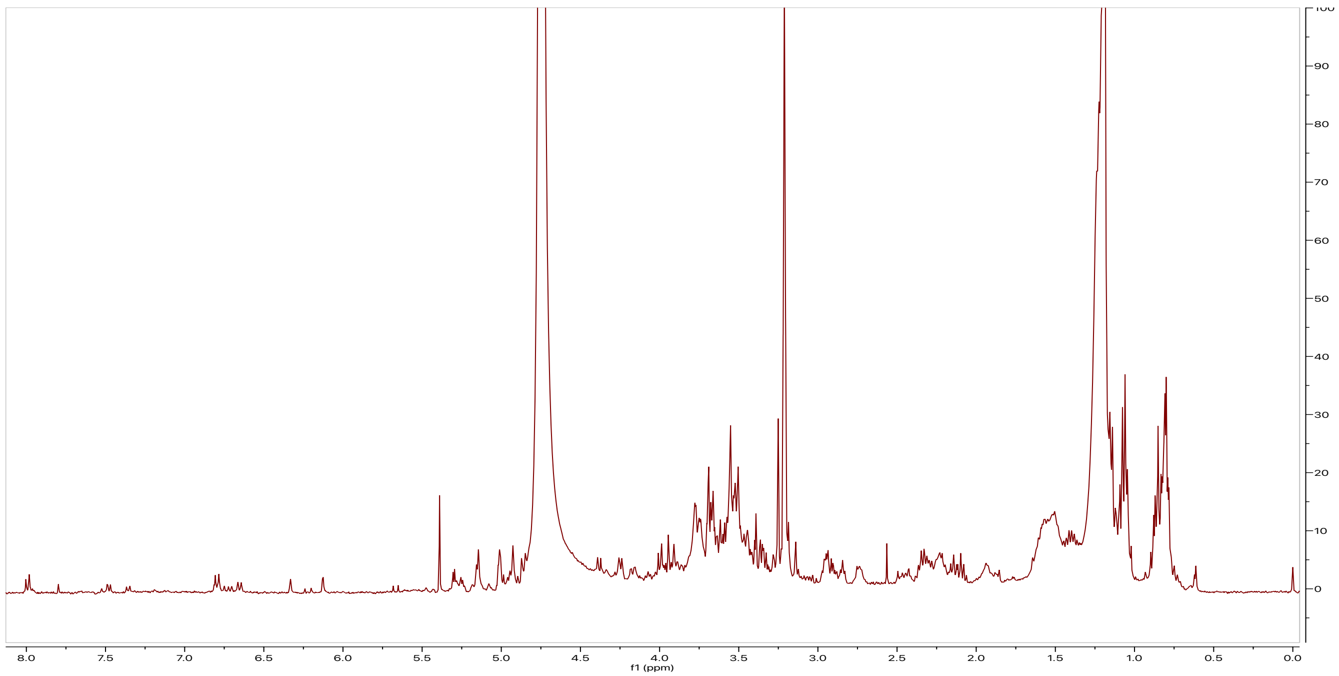

Figure S12. The ^{13}C NMR spectrum of fraction extract with rat intestine α-glucosidase enzyme in CD_3OD (125 MHz).

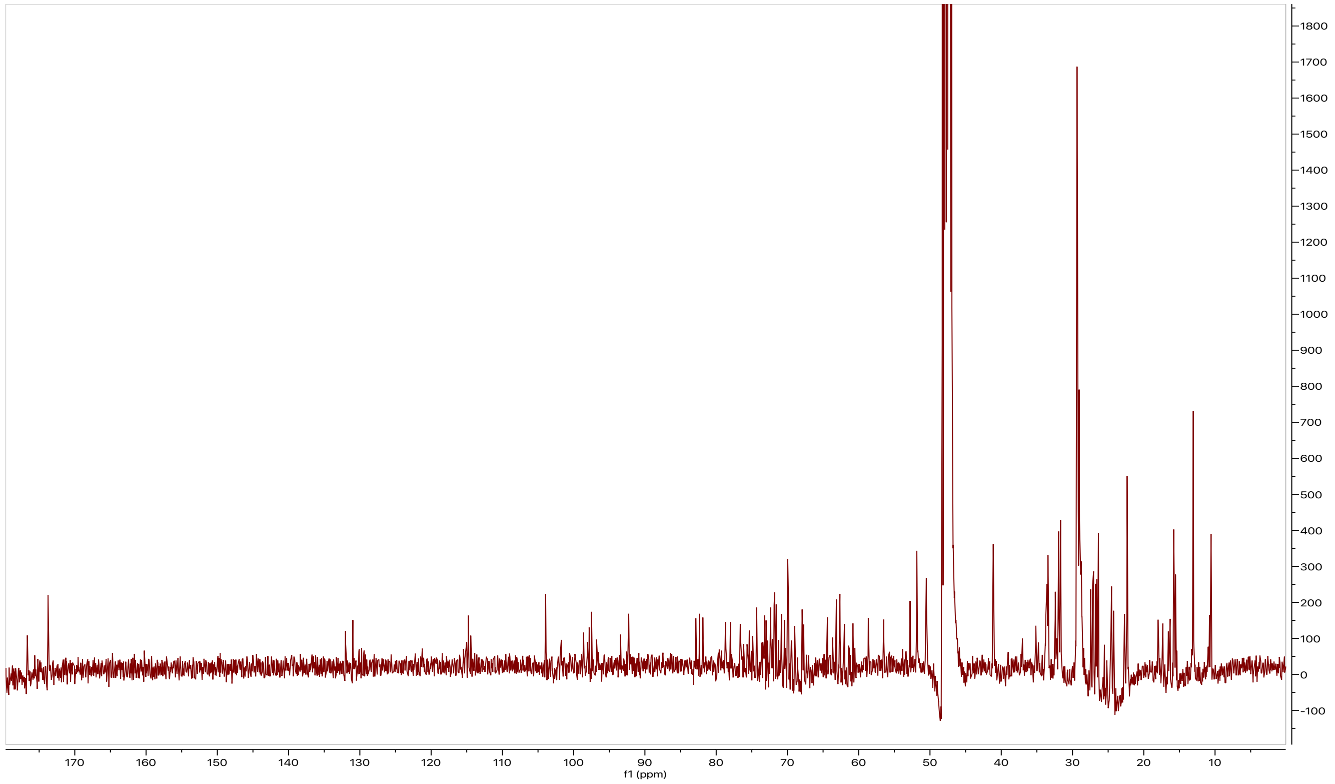

Figure S13. The HSQC spectrum of fraction extract with rat intestine α-glucosidase enzyme in CD_3OD.

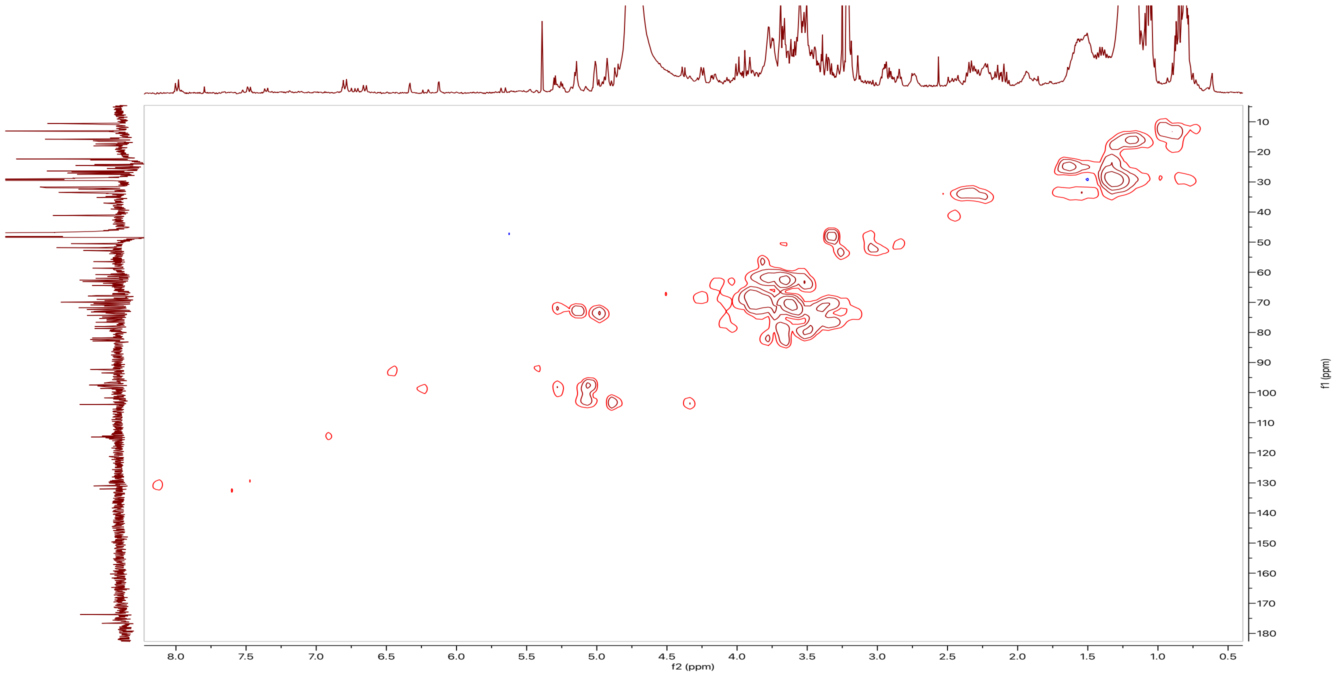

Figure S14. The HMBC spectrum of fraction extract with rat intestine α-glucosidase enzyme in CD_3OD.

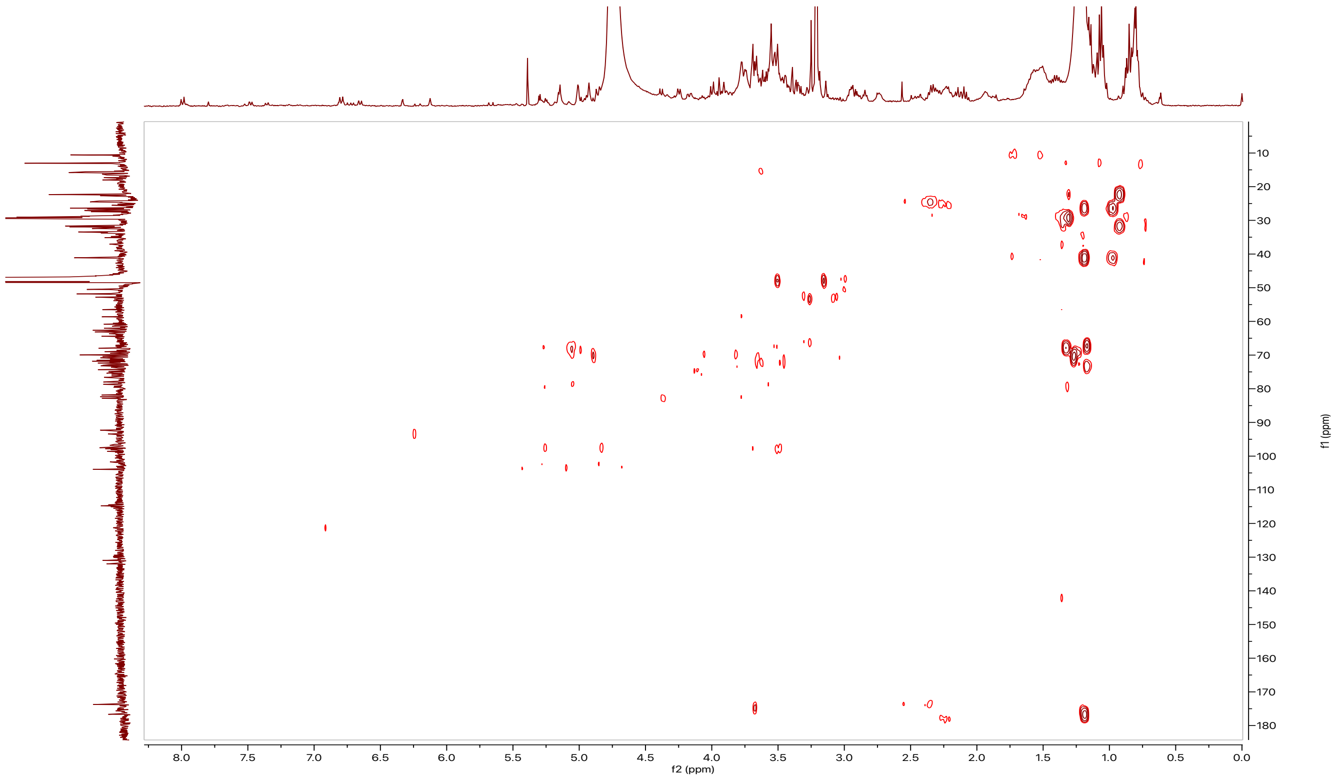

Figure S15. The ^{1}H NMR spectrum of fraction extract with hepatic G6Pase enzyme in CD_3OD (400 MHz).

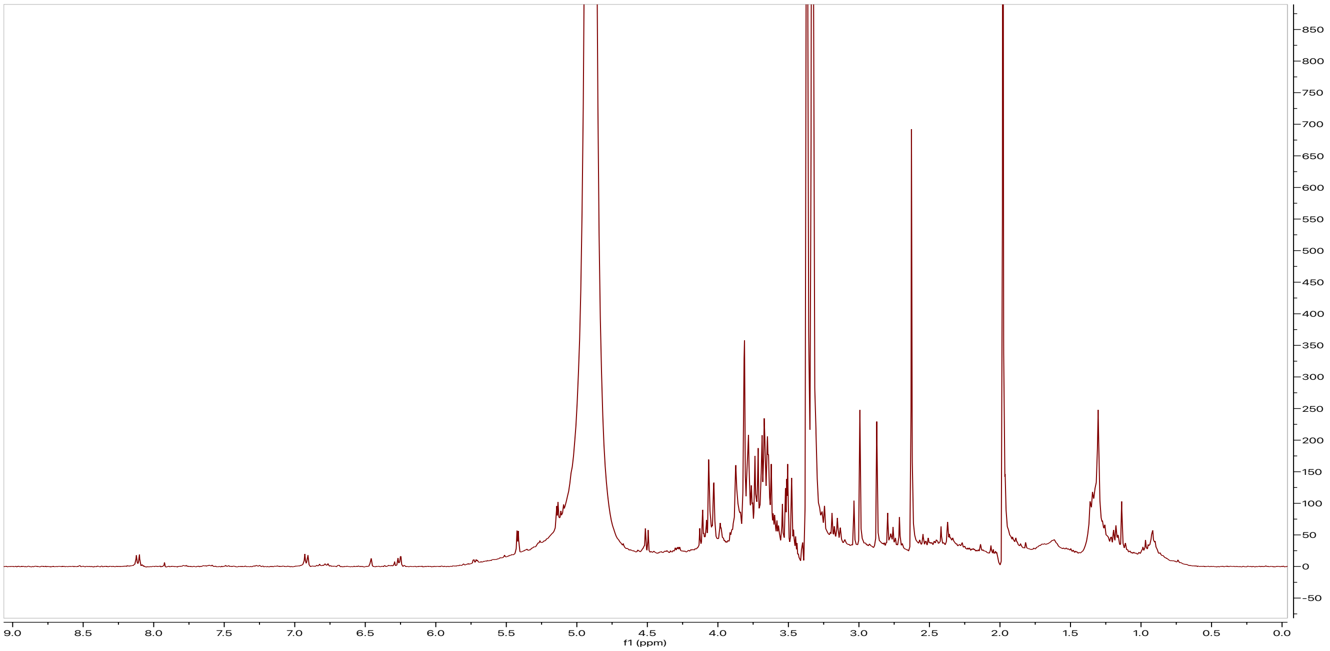

Figure S16. The ^{13}C NMR spectrum of fraction extract with hepatic G6Pase enzyme in CD_3OD (125 MHz).

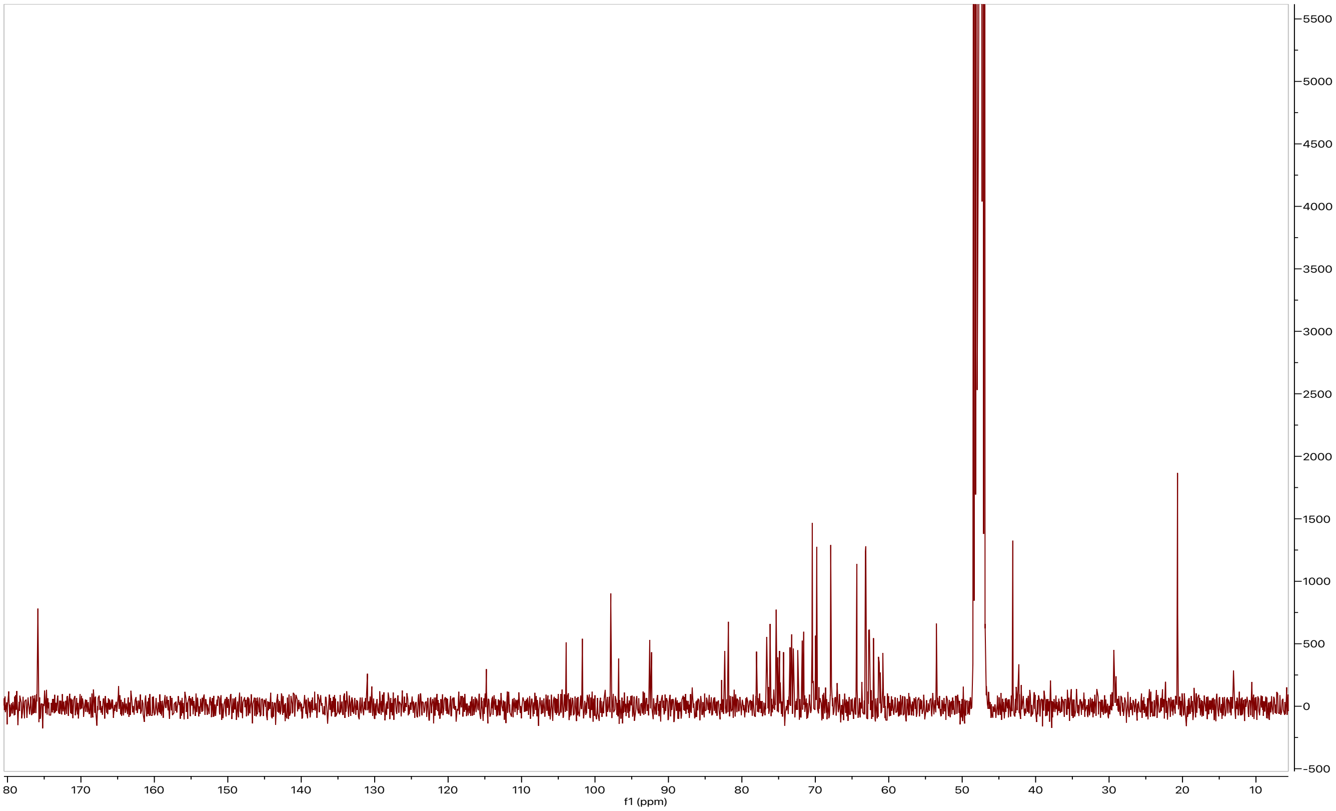

Figure S17. The DEPT90 spectrum of fraction extract with hepatic G6Pase enzyme in CD_3OD (125 MHz).

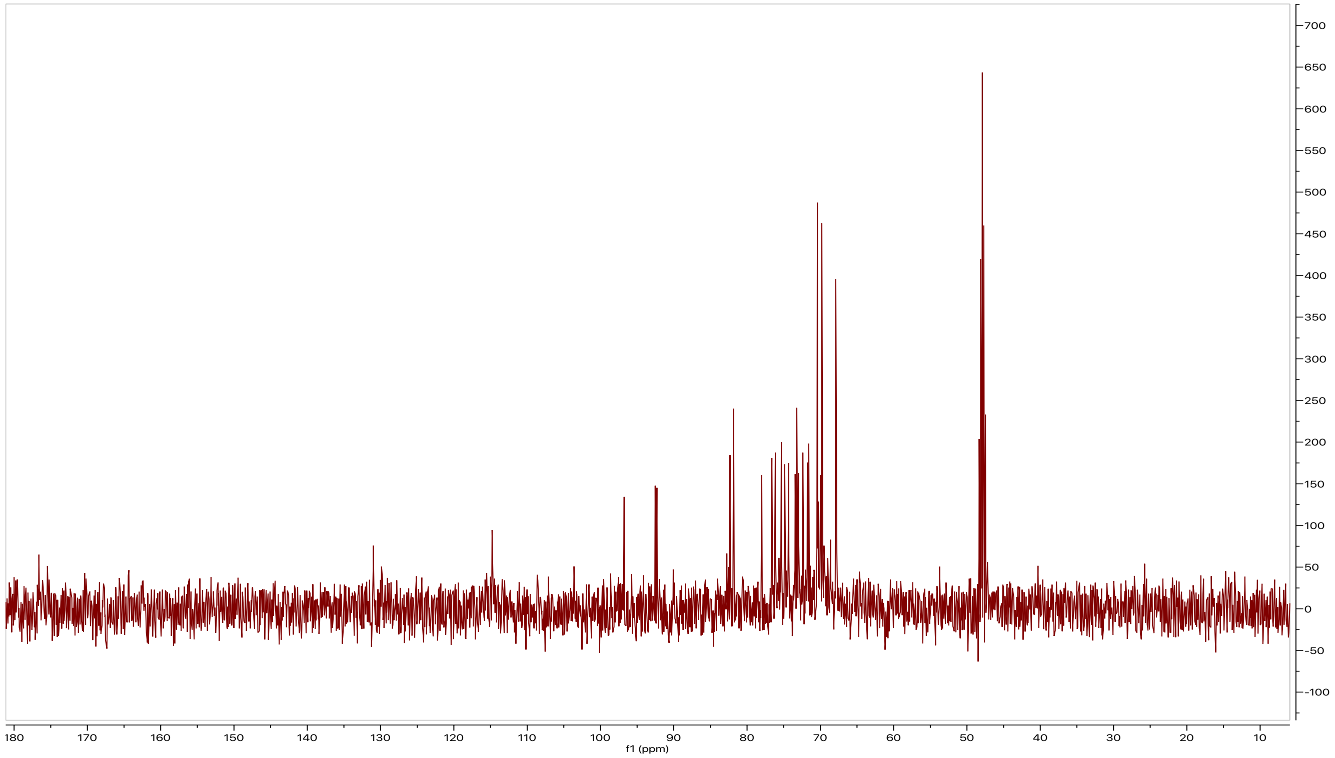

Figure S18. The DEPT135 spectrum of fraction extract with hepatic G6Pase enzyme in CD_3OD (125 MHz).

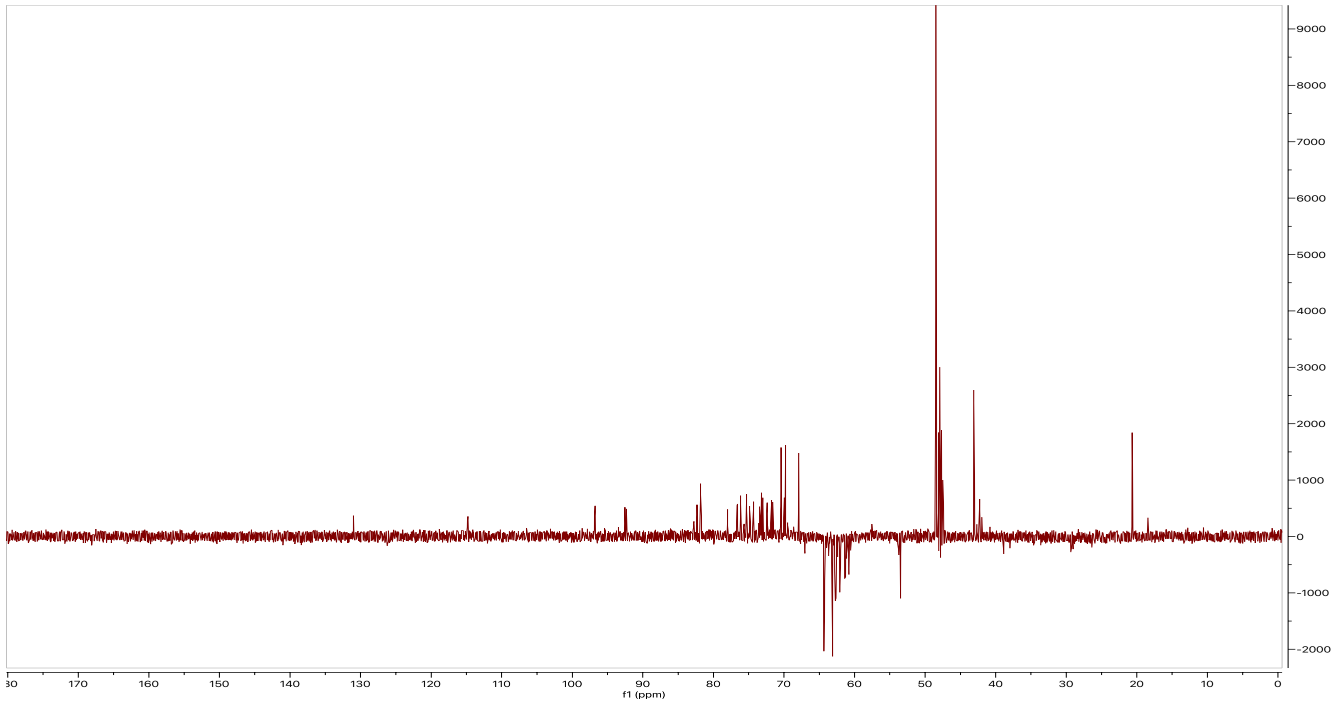

Figure S19. The HSQC spectrum of fraction extract with hepatic G6Pase enzyme in CD_3OD.

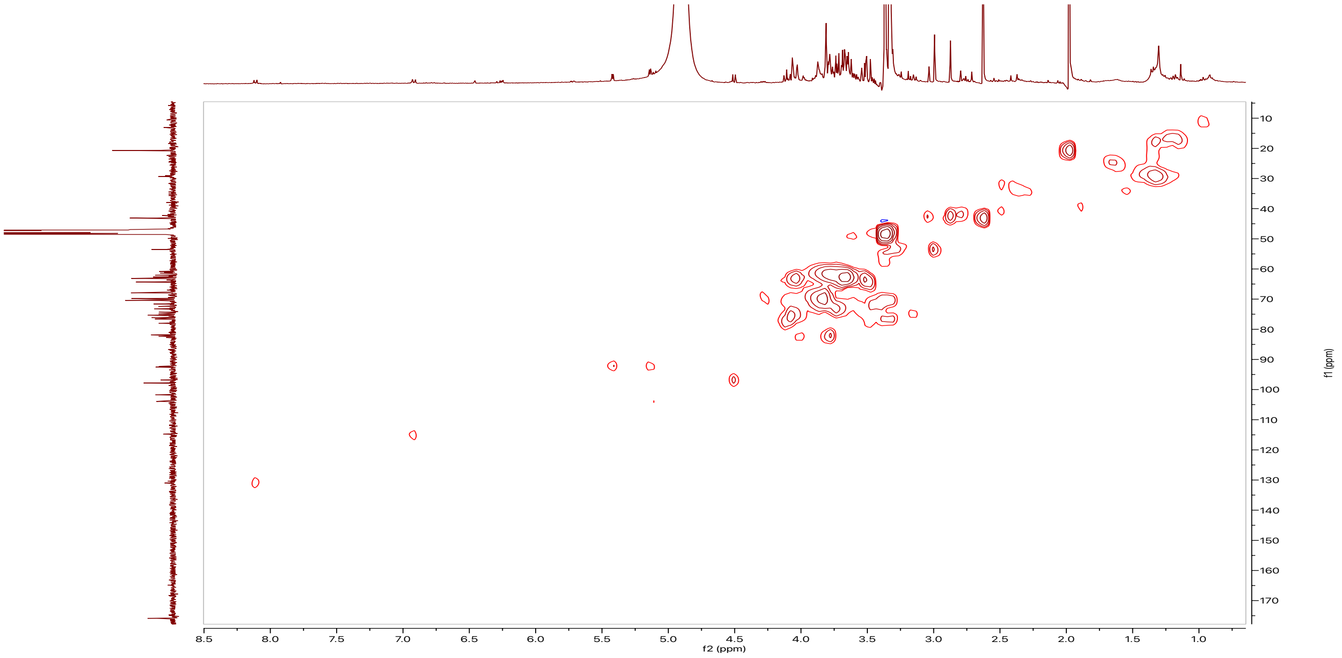

Supplement: Supplementary file 1 [file plants-13-00644-s001.zip › plants-2808854-supplementary.pdf]
